# Insights into Molecular Structure of Pterins Suitable for Biomedical Applications

**DOI:** 10.3390/ijms232315222

**Published:** 2022-12-03

**Authors:** Andrey A. Buglak, Marina A. Kapitonova, Yulia L. Vechtomova, Taisiya A. Telegina

**Affiliations:** 1 Faculty of Physics, St. Peterburg State University, 199034 St. Petersburg, Russia; 2 Institute of Physics, Kazan Federal University, 420008 Kazan, Russia; 3 Laboratory of Ecological and Evolutionary Biochemistry, Federal Research Center of Biotechnology, 119071 Moscow, Russia

**Keywords:** pteridines, photonics, computational chemistry, photosensitization, coenzymes

## Abstract

Pterins are an inseparable part of living organisms. Pterins participate in metabolic reactions mostly as tetrahydropterins. Dihydropterins are usually intermediates of these reactions, whereas oxidized pterins can be biomarkers of diseases. In this review, we analyze the available data on the quantum chemistry of unconjugated pterins as well as their photonics. This gives a comprehensive overview about the electronic structure of pterins and offers some benefits for biomedicine applications: (1) one can affect the enzymatic reactions of aromatic amino acid hydroxylases, NO synthases, and alkylglycerol monooxygenase through UV irradiation of H_4_pterins since UV provokes electron donor reactions of H_4_pterins; (2) the emission properties of H_2_pterins and oxidized pterins can be used in fluorescence diagnostics; (3) two-photon absorption (TPA) should be used in such pterin-related infrared therapy because single-photon absorption in the UV range is inefficient and scatters in vivo; (4) one can affect pathogen organisms through TPA excitation of H_4_pterin cofactors, such as the molybdenum cofactor, leading to its detachment from proteins and subsequent oxidation; (5) metal nanostructures can be used for the UV-vis, fluorescence, and Raman spectroscopy detection of pterin biomarkers. Therefore, we investigated both the biochemistry and physical chemistry of pterins and suggested some potential prospects for pterin-related biomedicine.

## 1. Introduction

**Figure 1:**
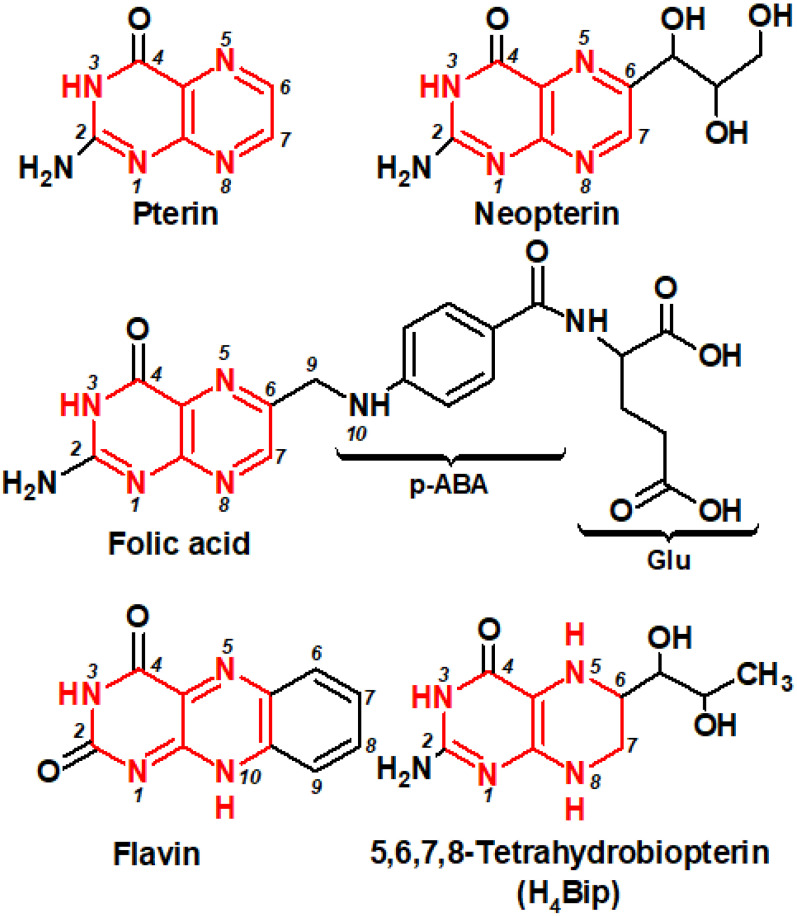
Chemical structure of pterins (pteridine system is shown in red).

Pterins are low-molecular weight heterocyclic compounds widely distributed in living organisms, primarily as reduced coenzymes. Structurally, pterins are a conjugated system of pyrazine and pyrimidine rings, the so-called pteridine, which is accompanied by a carbonyl group (C=O) at the C4 position and an amino group (NH_2_) at the C2 position (Figure 1). The pteridine structure is also characteristic of folates (folic acid and its derivatives) and flavins, or benzopteridines, which are derivatives of isoalloxazine. Folates are usually called “conjugated pterins” since they possess a para-aminobenzoilglutamine residue, whereas pterins are called “unconjugated pterins”. In addition to pterin, folates include a para-aminobenzoic acid (p-ABA) residue and one to five glutamic acid (Glu) residues. We will focus primarily on “unconjugated” pterins in this work.

The first known studies on pterins were started by Schopf et al. in the mid-1920s when leucopterin was discovered in whiteflies of the species *Pieris brassicae* and *Pieris napi* [1]. Since 1958, when Seymour Kaufman discovered 5,6,7,8-tetrahydrobiopterin (H_4_Bip), the biochemistry of H_4_Bip has been intensively studied [2,3,4]. At some point, in the 1970s–1980s the emission properties of pterins attracted the interest of analytical chemists and marine biologists [5,6,7]. The study of pterin photonics is a new field which arose in the late 1990s during the 20th century. Until that time, publications dealing with physical chemistry and the photonics of pterins were sporadic [8,9]. Systematic studies began with the emergence of two new research directions, both linked with molecular photonics. First, a pterin derivative, 5,10-methenyltetrahydrofolate (MTHF), was identified as a light-harvesting antenna, i.e., a participant in intermolecular non-radiation energy transfer, in the photoenzyme DNA-photolyase in a wide range of organisms, and also in the common regulatory photoreceptor cryptochrome [10,11]. It should be noted that the Nobel Prize in Chemistry was awarded to Aziz Sancar in 2015 for studying the mechanism of DNA repair by DNA-photolyases. Secondly, independently of these observations, a study of the basic photophysical and photochemical properties of biological pterins was started. It was found that (1) pterin molecules are active in electron transfer processes, including the oxidation of high-potential donors, which occurs with the participation of free radical forms [12,13]; and (2) the properties of excited pterin triplets were characterized and it was shown that pterin molecules are photogenerators of singlet oxygen with a quantum yield of up to 47% [14]. In subsequent works, the mechanisms of a number of photoinduced redox reactions involving excited pterins have been revealed [15,16,17]. Current research on the photochemistry of pterins is being actively carried out by the laboratory of Andres H. Thomas [18,19,20].

It has now become clear that the list of pterins involved in photoreception is not limited to MTHF, but also includes unconjugated pterins: cyanopterin [21] and 5,6,7,8-tetrahydrobiopterin (H_4_Bip) [22]. In studies of pterin photonics, the interest has been directed to reduced molecules [12,13,23,24,25,26] since reduced pterins predominantly function in the cell as cofactors of enzymatic reactions.

The participation of pterin coenzymes (reduced pterins) in photoreactions suggests their possible role as metabolic photoregulators. For example, the metabolic pathway of melanin biosynthesis, the initial stage of which is the enzymatic hydroxylation of phenylalanine (Phe) to tyrosine (Tyr), is H_4_Bip-dependent (Figure 1) [27,28]. The study of the photoprocesses of H_4_Bip and other pterins is of particular interest both for etiology and phototherapy of vitiligo (melanogenesis disruption). In this regard, a detailed analysis of the UV exposure effects on H_4_Bip oxidation is necessary [29,30].

Pterins are a class of photoreceptor molecules presented in a wide range of living organisms. The photonics of these compounds has been studied much less than the photonics of the universal chromophores, porphyrins, and carotenoids. The study of their electronic structure is important for the analysis of: (1) pterin photoreceptor functions in living organisms [21,31]; (2) the role of pterin coenzymes as regulators of enzymatic catalysis [32,33]; (3) pterins as photogenerators of singlet oxygen [34]. All of these aspects are significant from a biomedical viewpoint.

Therefore, the aim of this review is to analyze contemporary data on the physical chemistry and photonics of unconjugated pterins. We should answer the question: which properties of pterins are responsible for their photoreceptor functions and do they participate in energy and electron transfer reactions?

## 2. Different Oxidation States Relate to Different Biological Roles: Biochemistry In-Brief

Pterins are distinguished by the position and the nature of side-chain substituents: a variation of the substituent at the C6 position plays a paramount role (Figure 1). Furthermore, pterins differ by the degree of reduction as: (1) fully reduced, or tetrahydropterins; (2) semi-reduced, or dihydropterins; or (3) oxidized pterins. Tetrahydropterins, in particular, 6R-L-5,6,7,8-tetrahydrobiopterin (H_4_Bip) (Figure 1), play the role of key biological coenzymes.

### 2.1. Reducded Pterins

The H_4_Bip coenzyme is perhaps the most intriguing compound among unconjugated pterins and one of the most important coenzymes of higher organisms [28]. As an electron donor, H_4_Bip participates in the work of NO synthase (EC 1.14.13.39) [35]. As a reducing agent, H_4_Bip participates in the work of alkylglycerol monooxygenase [36] (EC 1.14.16.5) and hydroxylases of aromatic amino acids [37] [Fitzpatrick, 2003]: phenylalanine 4-hydroxylase (PAH; EC 1.14.16.1), tyrosine hydroxylase (TG; EC 1.14.16.2), and tryptophan hydroxylase (TPG; EC 1.14.16.4). H_4_Bip is transformed into the quinone 6,7-dihydro-L-biopterin (q-H_2_Bip) during the catalytic act in aromatic amino acid hydroxylases.

**Figure 2:**
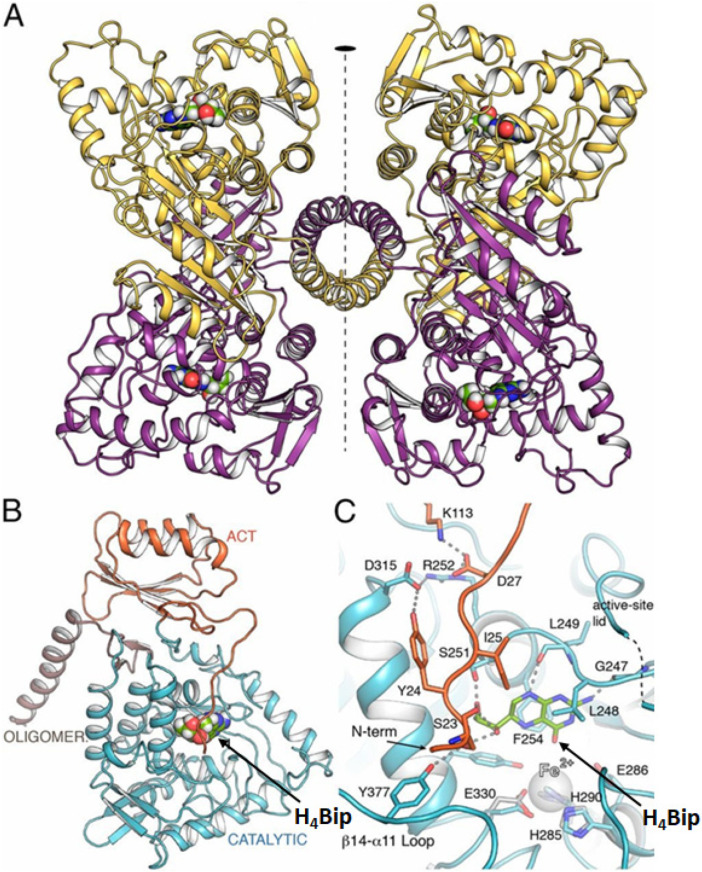
Crystal structure of hPAH. (**A**) The tetramer is formed as a dimer of dimers. Each protein monomer is stabilized by H_4_Bip. (**B**) The hPAH monomer with differential coloring of domains. (**C**) The active site of hPAH in complex with H_4_Bip. ©2019 National Academy of Sciences of the United States of America.

The PAH structure has been established with high resolution [38], and stabilization of the tetramer is caused by H_4_Bip binding. The structures of hPAH tetramers totally and partially bind with H_4_Bip (Figure 2) providing a rationale for H_4_Bip-responsive phenylketonuria by commercial H_4_Bip (sapropterin) and explaining the new stabilizing/chaperoning character of therapeutic approaches to address phenylketonuria. An excess of H_4_Bip oxidation products, oxidized pterins, in vivo can be a marker of various pathological processes [39]. Evidently, the same is true for the accumulation of H_4_Bip itself [40].

**Figure 3:**
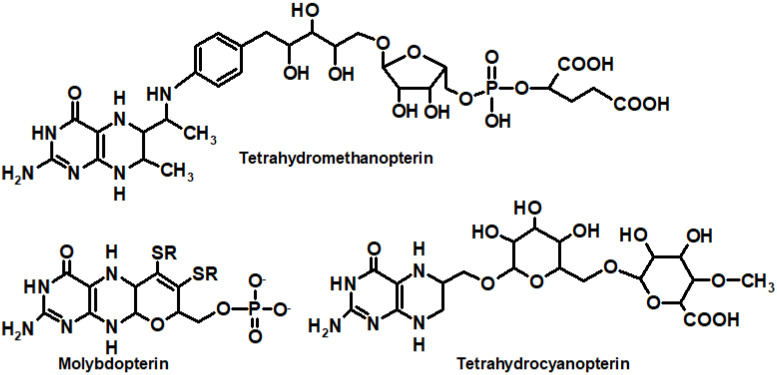
Chemical formulas of unconjugated tetrahydropterin coenzymes.

Unconjugated tetrahydropterins also include molybdopterin (which can exist in both the form of dihydro- and H_4_pterin), a coenzyme of xanthine oxidase, nitrate reductase, and several other enzymes [41,42]; tetrahydromethanopterin, a coenzyme of methanogenic bacteria [43]; and tetrahydrocyanopterin (Figure 3), recently discovered in cyanobacteria [44] and involved in the reception of ultraviolet radiation [45].

Methanogenic bacteria derive energy from the reduction of CO_2_ to methane. Methanopterin is a pterin derivative typical of methanogenic bacteria, which participates in carbon reduction reactions. In addition, 5,6,7,8-tetrahydromethanopterin is involved in a number of anabolic reactions [46].

The cyanopterin of cyanobacteria occurs in a concentration comparable to that of chlorophyll a (the molar ratio equals to 1:1.6). The in vivo oxidation state of cyanopterin is primarily the fully reduced 5,6,7,8-tetrahydrocyanopterin [44]. There is a hypothesis that cyanopterin can act as a chromophore of a putative UV-A/blue photoreceptor in cyanobacteria (see Section 7.1 for details) [21].

Molybdenum is a transition element and needs a special protein, molybdoenzyme, to be catalytically active [47]. The molybdenum cofactor (Moco) of molybdenum enzymes is composed of a molybdenum (Mo) coordinated by one or two molybdopterin ligands, called pyranopterins (Figure 3). The same pyranopterin cofactor is also known to coordinate a tungsten (W) atom in tungsten-containing enzymes. The pyranopterin ligand consists of (1) the pterin ring system, (2) the pyran ring conjugated with the pterin structure, and (3) the dithiolene moiety that coordinates the metal (Mo or W). Moco is conservative among living organisms, but the phosphate terminus is varied depending on the biological species: a CMP or GMP nucleotide can be attached [48]. Molybdenum enzymes participate in a variety of functions, from the global cycling of C, S, N, and As to prodrug metabolism [49].

Moco biosynthesis involves the subsequent cycle of reactions by six proteins and occurs in four stages, which require Fe and ATP [47]. Moco is distributed in an organism by respective proteins, and is unstable when dissociated from the protein [49]. Moco deficiency is known as type III xanthinuria, an inborn error of metabolism, and involves a deficiency of functional xanthine dehydrogenase and sulfite oxidase, which leads to the depletion of serum uric acid and the accumulation of sulfite due to the lack of molybdopterin [50]. Molybdenum cofactor synthase (MOCS1), molybdopterin synthase (MOCS2), and gephyrin protein (GPHN) are involved in Moco processing and are responsible for its deficiency [51]. Children with Moco deficiency have complex neonatal seizures, microcephaly, developmental brain abnormalities, and severe hypotonia. There is often a rapid decline that results in neonatal death. Their urine possesses a significantly elevated level of xanthine, hypoxanthine, and S-sulfocysteine [52]. Unlike other organic vitamins and cofactors, Moco cannot be taken directly as a food supplement, as it requires de novo biosynthesis [53].

Moco biosynthesis is essential for the virulence of several clinically important bacteria, including *Mycobacterium tuberculosis* and *Pseudomonas aeruginosa* [53]. Moco biosynthesis by enterobacteria in the gut microbiome is necessary for these organisms to cause inflammation; small molecule-inhibitors of Moco biosynthesis were efficient in preventing inflammation [54].

### 2.2. Semireduced Pterins

Dihydropterins are formed in vivo during enzymatic cycles. For example, the product of H_4_Bip oxidation, pterin-4a-carbinolamine, is oxidized to quinonoid-H_2_biopterin (qH2Bip) by pterin-4a-carbinolamine dehydratase (enzyme code (EC) 4.2.1.96) [55]. Dihydropterins are the substrates of key enzymes: sepiapterin reductase (EC 1.1.1.153), dihydropteridine reductase (EC 1.1.1.153), and dihydrofolate reductase (EC 1.5.1.3) [28]. DHPR is responsible for the following reaction:

6,7-dihydropteridine + NAD(P)H + H^+^ → 5,6,7,8-tetrahydropteridine + NAD(P)^+^ (Reaction 1).

**Figure 4:**
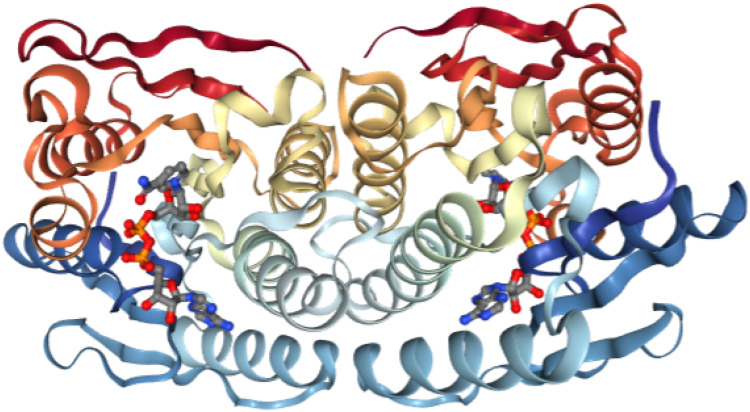
3D structure of human dihydropteridine reductase (1HDR). The monomers are stabilized by NADH molecules.

Thus, DHPR utilizes 6,7-H_2_pterin, NAD(P)H, and H^+^ to produce H_4_pterin and NAD(P)^+^. The 3D structure of the enzyme is presented in Figure 4. DHPR is a 26kDA alpha/beta protein with the Rossman fold for a dinucleotide coenzyme. DHPR is structurally and mechanistically distinct from dihydrofolate reductase, resembling NADH-requiring flavin-dependent enzymes [56]. An extra Thr residue after I22 in the human DHPR leads to DHPR deficiency, abnormal H_4_Bip metabolism, and H_4_Bip-associated diseases such as phenylketonuria. Cerebrospinal fluid analysis shows reduced concentrations of homovanillic acid and 5-hydroxyindoleacetic acid, decreased or normal H_4_Bip levels, and elevated dihydrobiopterin levels [57]. DHPR deficiency should be treated with H4Bip, Tyr, and DOPA, as well as low levels of Phe in a supplement.

DHFR is a small 24 kDa protein, which can reduce H_2_folate and H_2_Bip to H_4_folate and H_4_Bip, respectively [58]. The transfer of hydride from NADPH to H_2_folate occurs due to the conformational flexibility of Met20, which helps to stabilize the nicotinamide ring of NADPH and promotes the release of hydride [59]. DHFR deficiency causes megaloblastic anemia [60] and is treated with reduced forms of folic acid and folinic acid. DHFR mutations also result in pancytopenia, cerebral folate deficiency, and cerebral H_4_Bip deficiency, which can be treated with folinic acid [61]. Inhibition of DHFR as a therapeutic target with methotrexate and its analogues has been used for decades in cancer and bacteria treatment [62], since DHFR is responsible for dTMP biosynthesis. Trimethoprim (TMP) (2,4-diamino-5-(3′,4′,5′-trimethoxybenzyl)pyrimidine) is used as a template for the development of novel antifolate drugs against both Gram-positive and Gram-negative aerobic bacteria [63].

Sepiapterin reductase (SPR) is a homodimer composed of two subunits with a molecular mass of 28 kDa [64]. SPR uses NADPH and sepiapterin to produce NADP+ and 7,8-H_2_Bip and participate in H_4_Bip biosynthesis. SPR deficiency occurs due to SPR gene mutation and causes an inherited pediatric movement disorder called dystonia [65]. However, several other genes can be responsible for H_4_Bip-related dystonia [66]. SPR participates at the last stage of H_4_Bip biosynthesis, and the lack of H_4_Bip during SPR deficiency occurs only in brain, whereas other tissues are adopted to alternative paths of H_4_Bip synthesis. Other SPR-related diseases and symptoms include parkinsonian signs (tremor, bradykinesia, masked facies, rigidity), limb hypertonia, hyperreflexia, intellectual disability, psychiatric and/or behavioral abnormalities, autonomic dysfunction, and sleep disturbances [67]. SPR deficiency is treated with levodopa and carbidopa: carbidopa suppresses the peripheral metabolism of levodopa and it allows a great proportion of peripheral levodopa to cross the blood-brain barrier and affect the central nervous system. SPR can also play a role in chronic pain, cardiovascular disease, and cancer. Thus, SPR inhibitors can inhibit DNA synthesis and initiate the differentiation of erythroleukaemia (MEL) cells [64]. The list of SPR inhibitors include both natural and synthetic compounds: 6-carboxypterin (IC50 30 nM), rutin (60 nM), N-butyric acid (32 nM), dicoumarol (0.6 nM), etc.

### 2.3. Oxidized Pterins 

Oxidized pterins are present in living organisms mostly as oxidation products of tetra- and dihydropterins and are used in medicine as markers of oxidative stress [68], phenylketonuria [69], inflammation and activation of the immune system, cardiovascular diseases, neurotransmitter synthesis, and cancer [70,71,72,73,74,75].

The most common analytical methods of pteridine determination are high performance liquid chromatography (HPLC), capillary electrophoresis, and enzyme-linked immunosorbent assay (ELISA) [76,77]. HPLC can be used along with spectrophotometric, fluorescence, electrochemical detection, or mass spectrometry [78]. The particular biological fluids used for pteridine determination are blood serum [77,79], urine [73], and cerebrospinal fluid (CSF) [78]. 

**Figure 5:**
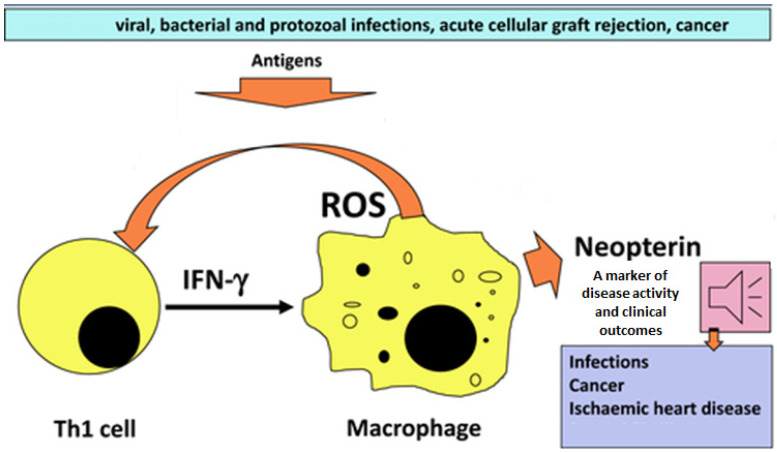
Neopterin (Nep) is involved in immune system activation and is a marker of disease activity in various inflammatory conditions. Nep, produced by activated macrophages in response to stimulation by interferon-γ, is a marker of immune activation [84].

Neopterin (Nep) is the product of 7,8-dihydroneopterin (H_2_Nep) oxidation. H_2_Nep is a potent antioxidant generated by macrophages, monocytes, and dendritic cells upon stimulation by gamma-interferon produced by T-lymphocytes (Figure 5) [80]. H_2_Nep protects macrophages from a range of oxidants through a scavenging that generates Nep or 7,8-dihydroxanthopterin. Thus, plasma and urinary Nep levels are dependent on macrophage activity [81]. This relationship has been clearly shown in studies of exercise and impact-induced injury during intense physical activity [82]. Urinary Nep and total Nep (Nep + H_2_Nep) levels are indicative of oxidative stress and trauma-induced inflammation [83].

Neopterin levels are sensitive to multiple diseases and pathological states, including even some exotic ones. For example, an elevated level of Nep in the cerebrospinal fluid was 100% sensitive for the diagnosis of cerebral malaria [85]. Serum Nep levels are nearly 10 times higher compared with healthy controls [86]. Serum levels of Nep are indicative of silicosis (a pathological state of lungs developed due to the inhalation of crystalline silica dust): the level of serum Nep in silicotic patients (24 nM) is twice higher than that of non-silica exposed patients (12 nM), and six times higher than that of the control group (4 nM) [87]. Moreover, Nep can be considered as a non-specific biomarker for inflammatory process in chronic obstructive pulmonary disease [88]. Blood Nep concentration is increased (>15 nM) in patients with pulmonary arterial hypertension and inoperable chronic thromboembolic pulmonary hypertension [89].

High Nep levels are indicative of atherosclerosis and other cardiovascular diseases. Nep has a crucial role in the atheromatous process and its useful in monitoring the severity of peripheral artery disease [90]. Nep is expressed at high levels in atheromatous plaques within the human carotid, coronary arteries, and aorta. The concentration of Nep is positively correlated with the plaque formation in carotid arteries in patients with atherosclerosis. H_4_Bip suppresses atherosclerosis and vascular injury and improves endothelial dysfunction. Evidently, the Nep production counteracts the progression of atherosclerosis. H_4_Bip and other Nep derivatives are a novel therapeutic target for atherosclerosis and other cardiovascular diseases [91].

Research on pteridines as urinary cancer biomarkers began in the mid-1980s [70,71]. Urinary pteridines are established as potential biomarkers in a host of diseases, including breast, prostate, kidney, and bladder cancers. There are 12 key pteridine cancer biomarkers: xanthopterin, isoxanthopterin, pterin, 6-biopterin, 7-biopterin, pterin-6-carboxylic acid, Nep, pterin, tetrahydrobiopterin, 6-hydroxymethylpterin, 6,7-dimethylpterin, and 6-methylpterin [92]. In a later study, elevated levels of urinary Nep, 6-biopterin, pterin, 6-carboxypterin, isoxanthopterin, and xanthopterin have been noted in patients with bladder cancer [93]. Isoxanthopterin specifically seems to be a compound that can be described as a biomarker of bladder cancer [75]. In ovarian cancer, elevated urinary Nep levels indicate an inflammatory reaction, which is cancer-determined [94].

The Nep concentration in the peripheral blood and in the tumor microenvironment correlates with phenotypic and functional changes of lymphocytes, indicating immune dysfunction [95]. The serum Nep levels of the patients with breast cancer (11.0 nM) were higher than those of controls (8.3 nM) [96]. Nep was significantly elevated in patients with advanced stages of breast cancer and grade III tumors. Metastatic disease was associated with significantly higher levels of Nep [97]. As a whole, serum Nep seems to be an indicator of metastatic cancer rather than a marker for local breast cancer [96]. Also, the serum Nep levels are indicative of prostate cancer [98].

The Nep level is elevated upon immune system activation in different types of cancer, including gastrointestinal ones [99]. Serological Nep is even indicative of gastrointestinal diseases. Serum Nep level is elevated during the pancreatitis. The monitoring of the serological Nep may be helpful for the prediction of the death risk from acute pancreatitis [100]. The Nep level may be a biomarker for osteoarticular changes of human brucellosis at an early stage [101]. The C-reactive protein and Nep serum levels are significantly higher in patients with gastric intestinal metaplasia and gastric atrophy: the best cut-off value to differentiate between patients with metaplasia and/or atrophy from controls was ≥10.15 nM for the Nep levels and ≥1.95 mg l^−1^ for the C-reactive protein levels [102]. Gastrointestinal Nep was elevated in COVID-19 patients compared with that in healthy controls. Moreover, patients with gastrointestinal symptoms had increased fecal Nep levels [103].

In general, CSF pteridines can be used as a biomarker of nervous system diseases. CSF Nep was significantly higher in patients with non-Hodgkin lymphoma compared with patients with predominantly peripheral infections, multiple sclerosis, or no disorder [104]. The CSF Nep concentration may be a good biomarker for the diagnosis, the monitoring of the disease course, and the prognostic evaluation of patients with primary central nervous system lymphoma [105]. The Nep level in CSF can serve as a biomarker in the diagnosis of human immunodeficiency virus (HIV) dementia, in the monitoring of the central nervous system’s inflammatory effects of antiviral treatment, and in giving valuable information on the cause of ongoing brain injuries [106].

The worldwide COVID-19 outbreak in 2020 led to multiple studies on Nep as a SARS-CoV-2 biomarker. It has been found that elevated Nep concentrations relate to a productive COVID-19 infection. A low or normal Nep is indicative of silent infection without or with less active virus production [107]. A high level of blood Nep (> 50 nm) is indicative of high fatal risks for patients with COVID-19 [108]. Nep is helpful for early prediction of COVID-19 severity and can serve as a prognostic marker [109]. Patients with COVID-19 do have neurologic symptoms, but the origin of central nervous system pathogenesis is unclear. The viral antigen was detectable in CSF and correlated with the immune activation of the central nervous system. COVID patients had markedly increased CSF Nep levels and signs of neuroaxonal injury [110].

Therefore, one can see that the content of oxidized pterins in biological fluids is indicative of a wide range of diseases. Nep is of particular interest for analytical determination, as well as 11 other oxidized pterins, which have already been mentioned.

### 2.4. Pterin Free Radical Species

All pterins can produce free radical species. Reduced pterin radicals are formed both enzymatically and non-enzymatically. Moreover, all classes of pterins can interact with enzymes involved in radical formation [111]. For example, H_4_pterins react with free radicals and serve as reducing agents. The most significant example of such interactions is NO synthase and its coenzyme H_4_Bip: the formation of H_4_Bip free radical derivatives (H_4_Bip^•+^/H_3_Bip^•^) is mandatory for normal NO production. H_4_Bip donates an electron during the NO formation and undergoes a one-electron redox cycle [112]. Pterins of all oxidation states are able to act both anti- or pro-oxidatively. In particular, reduced pterins, besides being scavengers of free radicals, are also strong reducing agents and they promote Fenton chemistry in the presence of transition metal ions. Oxidized pterins are known to be inhibitors or substrates of enzymes involved in free radical generation [113]. Therefore, it is necessary to consider the redox reactions of pterins in more detail.

## 3. Redox Chemistry of Pterins and Their Free Radical Species

**Figure 6:**
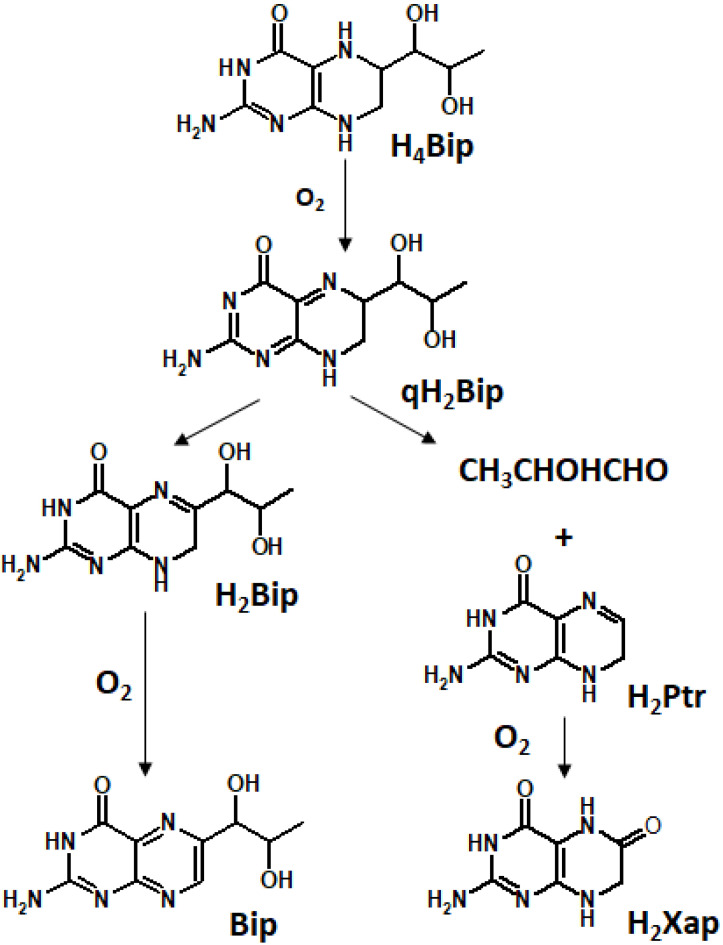
Scheme of H_4_Bip autoxidation in presence of molecular oxygen [26]. ©2014 Wiley.

It is well known that H_4_pterins, in particular H_4_Bip, are prone to autoxidation in the presence of O_2_ (Figure 6). In this process, superoxide anion radicals can be released. The direct reaction between H_4_Bip and O_2_ is an initiation reaction for the rapid reaction of O_2_^•−^ with H_4_Bip, very likely establishing a chain autocatalytic process involving the reduction of O_2_ by the intermediary tetrahydrobiopterin radical (H_4_Bip^•+^/H_3_Bip^•^) [114]. The respective reactions are as follows:

H_4_Bip + O_2_ → H_3_Bip^•^ + O_2_^•−^ + H^+^ (1)

H_4_Bip + O_2_^•−^ + H^+^ → H_3_Bip^•^ + H_2_O_2_ (2)

H_3_Bip^•^ + O_2_ → qH_2_Bip + O_2_^•−^ + H^+^ (3)

The rate constants of reactions 1, 2, and 3 are equal to 0.6 M^−1^ s^−1^, 3.9 × 105 M^−1^ s^−1^, and 3.2 × 103 M^−1^ s^−1^, respectively. According to quantum chemical calculations, the oxidation of H_4_Bip by free radical species and O_2_ occurs through the C8a atom, whereas electrophiles oxidize primarily the C4a-N5 site of H_4_Bip [30]. The latter is in agreement with the enzymatic oxidation of H_4_Bip to pterin-4a-carbinolamine [115].

Detection of quinonoid 6,7-dihydrobiopterin (qH_2_Bip) is a challenging task since it quickly transforms to a more stable 7,8-dihydrobiopterin (H_2_Bip). Thus, integration of an infrared photodissociation spectroscopy along with liquid chromatography–tandem mass spectrometry is required [116].

The chemistry of pterin free radical species largely determines their physiological role and biochemical functions. For example, the electron donor properties of H_4_Bip and its free radical derivatives play a key role in the production of NO, an important cellular signaling molecule, which, in particular, modulates the vascular tone [112,117]. 

H_4_Bip can spontaneously produce ROS as well as scavenge them. This allows H_4_Bip to regulate the ROS levels in the endothelium [118]. It makes it possible to use H_4_Bip as a therapeutical agent in cardiovascular medicine. As a whole, oxidation of H_4_Bip in oxygenated solutions occurs according to the following equation:

H_4_Bip + O_2_ → qH_2_Bip + H_2_O_2_ (4)

Free radical species may lead to the formation of a peroxide form of H_4_Bip. However, according to later evidence, the equilibrium of the following equation is shifted to the left side [114]:

H_3_Bip^•^ + O_2_^•−^ → H_3_BipOOH (5)

The termination reaction of H_4_Bip autoxidation is a dismutation act [114]:

2H_3_Bip^•^ → H_4_Bip + qH_2_Bip (6)

As one can see, the autoxidation of H_4_Bip as a whole is a chain free radical process. In the presence of light, it transforms into the photo-oxidation, which is also a free radical chain process, but also with an autocatalytic character [26,30]. 

H_2_Nep is also an important ROS scavenger. While peroxyl and hydroxyl scavenging generates dihydroxanthopterin (H_2_Xap), hypochlorite efficiently oxidizes H_2_Nep into Nep [119]. Superoxide reacts with H_2_Nep, which results in Nep formation. H_2_Nep reacted with O_2_^•−^/OH^•^ mixtures generated by X-ray radiolysis to Nep [119]. Production of Nep by O_2_^•−^ obtained from the xanthine/xanthine oxidase system was inhibited by superoxide dismutase. Therefore, H_2_Nep scavenges superoxide and is subsequently oxidized into Nep in cells and cell-free experimental systems.

Moreover, all classes of pterins (tetrahydropterins, dihydropterins, oxidized pterins) can participate in radical mediated reactions [111]. All the classes have been shown to act as both pro- and antioxidants. Oxygen-, nitrogen-, and pterin-radicals of H_4_pterins are formed enzymatically and non-enzymatically. H_4_pterins interact with free radical species and reduce them. The net effect of pro- and antioxidant activity of a particular compound is a question of the experimental settings, whereas the particular physiological role often remains unclear [111].

During the catalytic act of NO synthase, H_4_Bip undergoes a one-electron redox cycle [112,120]. The binding of H_4_Bip is essential for NO synthesis by NO synthase (NOS) enzymes: H_4_Bip plays the principal role of electron donor in the catalytic cycle of NOS. Other pterins are either unable to support NO synthesis in NOS enzymes, or can only support a much slower NO synthesis rate than H_4_Bip [121]. H_4_Bip can be oxidized in vivo by O_2_ or ROS to generate H_2_Bip, a pterin structurally similar to H_4_Bip but unable to support NO synthesis. H_4_Bip homeostasis determines the role of NO synthase, affecting the production of nitric oxide and ROS. Another interesting aspect is that the H_4_Bip/NOS ratio may regulate cellular radiosensitivity, therefore, it is possible to control radiosensitivity through H_4_Bip metabolism [122].

Thus, H_4_Bip itself is highly susceptible to oxidation. H_4_Bip autooxidation occurs spontaneously in aqueous solutions in the presence of O_2_. In general, the process of H_4_Bip autoxidation has a chain radical character [114] and the influence of H_4_Bip autoxidation on physiological processes can hardly be overestimated. For this reason, precise determination of H_4_Bip is much needed both in aqueous solutions and biological fluids [123].

## 4. Computational Studies

Computational and theoretical chemistry studies of pterins in the mid-1990s were started by the group of G. Reibnegger in a series of publications on H4Bip conformational flexibility. Using ab initio calculations, they established that the axial conformation is more stable than the equatorial one by 2.2 kcal mol−1. This was in agreement with a molecular dynamics simulation on a picosecond timescale. The axial conformation is stabilized by two intrinsic hydrogen bonds between the pyrazine ring and the side chain, whereas the equatorial conformation has a single H bond [124]. This conclusion was supported later by NMR [125] and density functional theory (DFT) calculations [126]. In yet another study, a comparison between conformational flexibility of H4Bip and H4Nep has been made. It was shown that both biologically active (6R,1′R,2′S)-5,6,7,8-tetrahydrobiopterin and biologically inactive (6R, l’S,2′R)-5,6,7,8-tetrahydroneopterin prefer the axial conformation. Therefore, their different biological activity cannot be explained by different conformational properties [127]. One should also take the equatorial conformation into account when studying H4Bip since it is close to the axial one and transforms from one to another in several picoseconds. This series ends with a publication in which a simple error backpropagation neural network (NNet) was applied (long before the machine learning boom in the 2010s) to the conformational space created by two torsional angles, N5-C6-C1′-C2′ and C6-C1′-C2′-C3′. The application of NNet helped to simplify the scanning of relaxed potential energy surfaces.

Electronic structure investigation of H4Bip and three of its analogues revealed the characteristics responsible for the NO synthase inhibition by 4-amino-H4pterin [128]. Differences in electron density, the Mulliken charge, and electrostatic charge distribution are responsible for the different activity of H4lumazine, H4pterin, and 4-amino-H4pterin. The electron density peculiarities of H4lumazine are located at N1 and C2 position, whereas 4-amino-H4pterin totally differs from H4pterin and H4lumazine, especially at N3.

**Figure 7:**
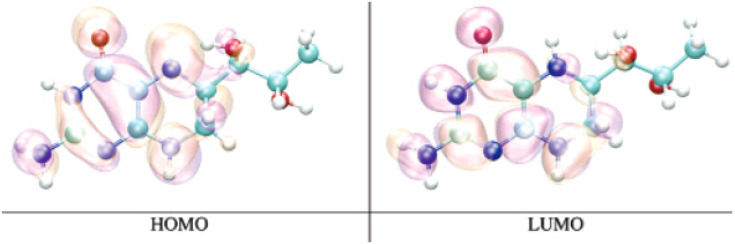
Frontier molecular orbitals of H4Bip [126]. ©2006 American Chemical Society.

V. Gogonea et al. were the first known researchers to study the electronic structure of pterins, in particular, H4Bip [126]. From the depicted HOMO, LUMO (Figure 7), and SOMO orbitals for neutral H4Bip, its cation and anion, the values of the ionization potential (IP), and the electron affinity (EA) in the gas phase, water, and protein environment were found. Thus, the neutral H4Bip is the most stable in gas, water, and protein environments, whereas in a dielectric environment an anion becomes the most stable species. The IP of H4Bip in proteins is equal to nearly 0.5*IP in the gas phase, and its EA is about 0.2*EA in the gas phase. The amino acid movement around H_4_Bip may lead to configurations where H_4_Bip^•-^ anion is more stable than the neutral H_4_Bip, which facilitates electron transfer and redox reactions of H_4_Bip as a biological coenzyme.

**Figure 8:**
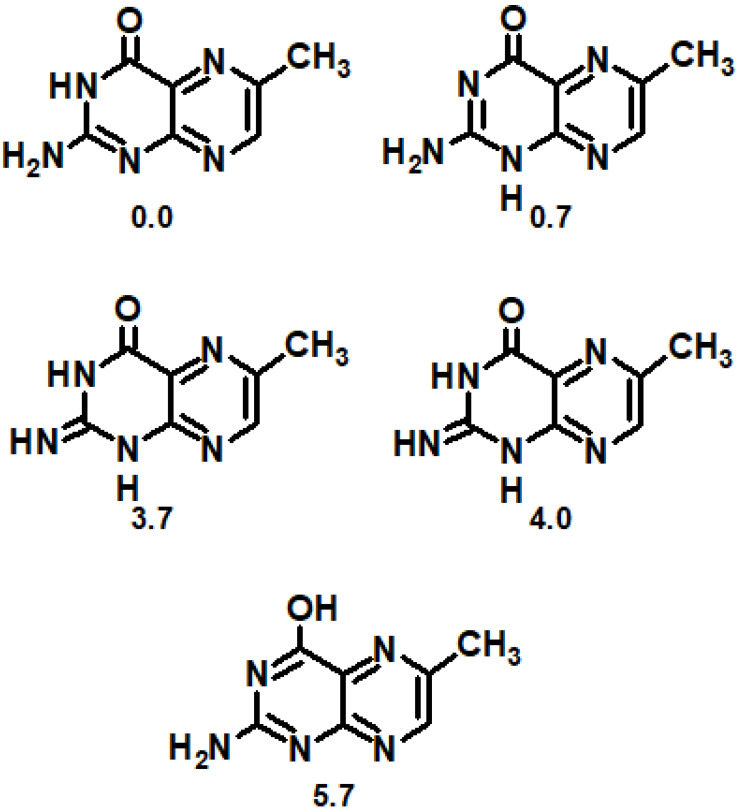
Low-energy tautomers of 6-methylpterin (6-Mep) with relative energies (in kcal mol^−1^) according to the B3LYP/6-31G(d,p) method [129].

What about dihydropterins and oxidized pterins? Regarding oxidized compounds, tautomer analysis of pterins reveal the existence of three additional low-energy tautomer forms (Figure 8) with a relative energy ≤4.0 kcal mol^−1^ apart from a well-known lactim tautomer (5.7 kcal mol^−1^) [129]. All five tautomers are significant for biological systems. Regarding anion tautomers, it was shown that all lactim forms do possess ΔE more or equal to 22 kcal mol^−1^, which makes them biologically insignificant [130]. Apart from lactam, only one more tautomer was biologically significant: N3H, N9H (1.7 kcal mol^−1^). Regarding cations, it was found that the sites primarily responsible for proton attraction were N1 (0 kcal mol^−1^), N8 (1.2 kcal mol^−1^), and N5 (3.7 kcal mol^−1^) of the lactam, followed by N1 of the lactim (4.7 kcal mol^−1^) [131]. 

Bader’s atoms-in-molecules quantum theory was applied to pterin, its anion, and cation [132]. The bond orders of the pyrimidine ring change upon ionization and de-ionization. Neutral pterin shows a negative electrostatic potential along the axis of O4 and N5, along N3 and N8. Such studies give fruitful information about the electronic structure, electrostatic mapping, and biological activity. A study of the Fukui indices and individual reactivity of atoms in H_4_Bip has been also performed. It was found that N5 is mostly responsible for the interaction with the electron acceptor, whereas C8a is the primary center of attack by the radical species [30]. 

In another study, the quantum theory “Atoms-in-Molecules” (QTAIM) analysis of radical species was conducted. The radicals obtained through the detachment of N-bonded H atoms were more stable in comparison with radicals obtained through the C-H repulsion. N-centered radicals showed significant delocalization of spin density over both pyrazine and pyrimidine [133]. N3 and N9H radical forms were the most stable in the gas phase and water, respectively. 

Calculations for various pteridine compounds, both oxidized and reduced, revealed lumazine as the molecule with the highest oxidation potential (56.4 kcal mol^−1^) [134]. The reason, obviously, being the presence of two carbonyls in the pyrimidine ring. Among oxidized pterins, 6-formyl-pterin (Fop) possesses the highest oxidation potential (47.5 kcal mol^−1^) because of the carbonyl C6-substituent [135]. Interestingly, quinoid dihydropterins as well as 6,7-dihydropterin, show oxidation potentials in the same range as oxidized pterins. On the other hand, quinonoid tautomers and 6,7-dihydropterin are more easily reduced than other dihydropterins. In the course of enzymatic reaction, H_4_Bip is oxidized and needs to be regenerated. The high oxidation potential of the quinoid prevents further oxidation to biopterin, while the smaller reduction potential facilitates the reduction to H_4_Bip. The hydroxyl radical formation by 10 dihydropterins was investigated in a joint theoretical-experimental study using quantitative structure-activity relationship (QSAR). 7,8-dihydro-6-methylpterin (H_2_Mep) showed the highest rate constant of OH^•^ formation, whereas sepiapterin possesses the lowest one. The intensity of OH^•^ formation correlated with the oxidation potential of pterins and the side chain nature at the C6 position [136]. Alkylation and carbonylation (sepiapterin) favor the opposite properties.

The alkylation of pterins enhances their ability to interfere and cross membranes, which is important when interacting with cells. Methylation of Mep (isomers and energy barriers) was investigated with DFT. The N-methylated isomer is 6.8 kcal mol^−1^ more stable than the O-methylated one. The energy barrier for the O- to N-alkylated isomer rearrangement equals 53.2 kcal mol^−1^, so the authors conclude that these isomers are non-interconverting [137].

Regarding photochemistry, pterin alkylation lowers the triplet state energies, which is consistent with enhanced ^1^O_2_ quantum yields. Pterin alkylation at O4 or N3 lowers T_1_ by 0.4–3.6 kcal mol^−1^ as compared to Ptr [137]. Moreover, alkylation at O4 leads to the lower excited state energies as compared to N3 alkylation: O-alkylated pterin has a S_0_→S_1_ transition equal to ~76 kcal mol^−1^ and a S_0_→T_1_ transition equal to ~64 kcal mol^−1^. In contrast, N-alkylated pterin has a higher S_0_→S_1_ (~81 kcal mol^−1^) and a higher S_0_→T_1_ transition (~68 kcal mol^−1^). We may conclude that O-alkylation is more prospective than N-alkylation in terms of the higher ^1^O_2_ quantum yield. On the contrary, alkylation lowers the fluorescence quantum yield Φ_fl_ from 33% of Ptr to 12% and 7.8%, respectively [138].

The works on pterin computational photonics [139,140,141,142] will be considered in detail in Section 5.2, Section 5.3 and Section 5.4. Interactions with metals significantly influence the electronic properties of pterins and are relatively easy to be obtained. For this reason, the next section is dedicated to the interactions with metals.

## 5. Interactions of Pterins with Metals

Consideration of pterin-metal interactions is important from a biomedical point of view since, in living systems, pterins usually function in complex with metals, and in order to decipher, for example, the mechanism of an enzymatic pterin-dependent process, it is necessary to understand the regularities of pterin-metal interactions. Pterins, as molecules with planar heterocyclic rings, are capable of forming stable complexes with ruthenium. The fluorescence spectra and electrochemistry of complexes of ruthenium with various pteridine derivatives (lumazine, 1,3-dimethyllumazine, 3-methylpterin, 8-methylpterin, and 3,6,7-trimethylpterin) have been studied [143]. It has been shown that chelation with ruthenium(II) at N5 and O4 results in intense metal-ligand charge-transfer (MLCT) transitions near 600 nm and coordination-induced shifts of the π→π* transitions in ligands between 330 and 430 nm, and in the general case, the pterin fluorescence excitation bands are shifted towards higher energies. This is explained by the formation of retrodative bonding between the ruthenium d orbitals and π* orbitals of aromatic ligands involving nitrogen.

Pterin complexes with ruthenium(III) are able to participate in proton-coupled electron transfer (PCET) as an acceptor molecule [144]. In such a reaction, the pterin ligand behaves as a proton acceptor, whereas the Ru(III) metal center acts as an electron acceptor. It has been shown how the complex acts as a PCET acceptor from O-H [145] and C-H [146] bonds.

Ragone F. et al. have managed to obtain and characterize complexes of pterin with rhenium(I) [147,148]. Interestingly, the Re(CO_3_)(pterin)(H_2_O) complexes are highly soluble in water, although pterins and their constituents are usually soluble only in organic solvents. The Re(I) ion turned out to be in a slightly distorted octahedral environment and is coordinated by the planar pterin molecule at the O4 and N5 binding sites, as was observed with ruthenium and other metals. It has been shown from the absorption spectra that the Re(pterin) complex is stable at pH ranging from 2 to 11. Some of the observed changes in the dominant absorption bands are explained by contributions from different states of the ligand, inter-ligand transitions, and metal-to-ligand charge-transfer transitions. For the protonated states of the Re(CO_3_)(pterin)(H_2_O) complex, pK_a1_ = 3.9 and pK_a2_= 8.8 were calculated. Similar metal complexes for pterin derivatives were also described in the case of coordination with iridium(III) [149].

Another interesting aspect linking pterins with platinum group metals (Ru(III), Os(VIII), Pd(II), and Pt(IV)) was the ability of metal ions to catalyze the oxidative conversion of folic acid to pterin-6-carboxylic acid, para-aminobenzoic acid, and glutamic acid by sodium N-bromo-p-toluene-sulfonamide (bromamine-T, or BAT) [150]. Despite the similarity of these metals, Kumar et al. write that the mechanisms of their catalytic activity are different. A study of the reaction kinetics shows that metal ions accelerate the process of oxidative conversion of folic acid by an average of 6–22 times.

**Figure 9:**
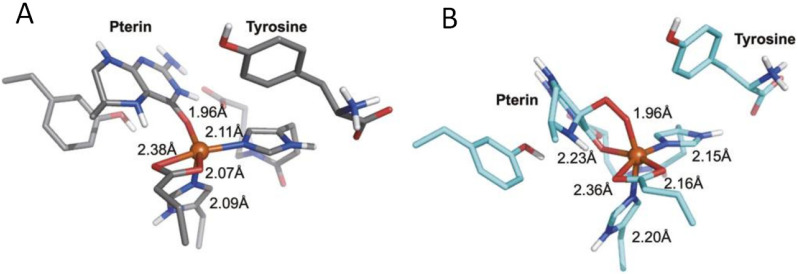
Structures of the human tryptophan-hydroxylase ternary complex (**A**) and the peroxy intermediate with the pterin carbonyl bound (**B**). The metal–ligand distances are indicated. © 2022 National Academy of Sciences of the United States of America.

Pterin-dependent nonheme iron monooxygenases hydroxylate aromatic amino acids, which are the precursors of neurotransmitters biosynthesis; this supports the normal brain functioning. The normal functioning of these enzymes requires the presence of an iron atom and tetrahydrobiopterin (H_4_Bip) in its active center [151]. Using the example of the human tryptophan-hydroxylase (TPH) protein, it was shown that a ternary complex [152] is formed between the pterin cofactor, iron(II), and the substrate, tryptophan (Figure 9). With a metal center, the pterin donates two electrons for O_2_ activation. This significantly reduces the energy barrier of the reaction, which can be described as follows [152]:

(Fe(II)/H_4_Bip/Trp)-TPH + O_2_ ⇌ (Fe(II)/H_4_Bip/Trp)-TPH-O_2_ → Intermediate → Fe(IV)=O + H_3_BipOH → Fe(II) +Trp-OH +H_2_Bip (7)

By analogy with such iron-containing complexes, similar double and triple complexes of pterins and their derivatives with copper were studied. The reason for studying such interactions was that Cu^2+^ was found in PAH from *Chromobacterium violaceum*, and it was suggested that copper is involved in the catalytic activity instead of iron [153], which was later questioned [154,155]. Nevertheless, it was shown that the same O4 and N5 serve as the main binding sites for copper with pterin and its derivatives. Triple complexes of different constituents have been studied, for example, Cu(II) complexes Cu(DA)(Ptr), where DA = 2,2’-bipyridine (bpy), 1,10-phenanthroline (phen), or ethylenediamine (en) and Ptr = folic acid (FA), lumazine, or related compounds [156]. In some studies, 6-carboxypterin was chosen as a model pterin, for example, ([Cu(bpy)(Cap)(H_2_O)] [157].

Nickel and cadmium ions and their compounds are often toxic to living organisms; therefore, their interaction with nucleic acids, proteins, and other biological molecules is being studied. As in many previously described cases, pterin behaves as a bidentate ligand capable of binding a metal ion at O4 and N5, forming a five-membered ring with it. When compared with binding constants, the most preferable Ni(II) chelation occurs in the following order: pterin > 6-carboxypterin > folic acid, which are differed from each other by the side chain at C6. The study of the kinetics of the chelation reaction suggests that from the mixing of pterin with metal ions until the formation of the bidentate complex, there is some transitional monodentate state, and the chelation process itself is controlled by deprotonation of the OH group of the pterin. Crispini A. et al. reported self-assembled and self-organized octahedral pterin complexes coordinated by Ni(II) and Cd(II) [158]. In general, such complexes are defined by the formula [M(en)(Ptr)_2_], where M = Ni or Cd, en = ethylenediamine, and one metal ion coordinates two pterin molecules simultaneously. These complexes turned out to be highly soluble in aqueous solution. Deciphering the crystal structure showed that the metal-supramolecular system is formed due to a set of hydrogen bonds O-H- - -O, N-H- - -N, and N-H- - -O types and π-π interactions.

Another area of research is the study of artificial systems of pterins and their derivatives with noble metals due to the widespread development of nanotechnology and new methods for detecting organic compounds. The first to study the interaction of pterins (pterin, isoxanthopterin and sepiapterin) with Cu, Ag, Au, Zn, Cd, and Hg metal atoms in terms of electron donor–acceptor properties using density functional theory (DFT) were A. Martinez and R. Vargas [159]. Neutral metal atoms in a gaseous medium and cations and dications in an aqueous medium were considered. It was shown that among the neural atoms, only Cu is able to bind with pterins. Metal cations and dications strongly bind to pterins in all cases, modifying their electron donor–acceptor properties. The complexes with Cu, Ag, and Au cations proved to be good electron acceptors, and the complexes with Zn, Cd, and Hg—electron donors. Only in the case of Zn, Cd, and Hg cations is an exergonic reaction with HO· possible when the calculated adiabatic Gibbs free energy ΔG° < 0.

The same authors have studied complexes of pterins with metal anions and negatively charged clusters [160]. They have studied the interaction between Cu, Ag, and Au anions and the three pterins (pterin, isoxanthopterin, and sepiapterin). It has been shown that non-conventional hydrogen bonds are formed between the N-H groups of pterins and metal atoms. In all stable structures, two H bonds were formed between the pterin and the metal. The bonds between the metal and pterin were shorter with Au than in the case of the other metals, which indicates that these bonds are stronger. In addition, [(7-Xap)-Me]^−1^ complexes were found to be more stable than [Sep-Me]^−1^ and [Ptr-Me]^−1^. The authors argue that the main contribution to the formation of non-conventional H bonds is made by the electrostatic attraction between the metal anion and the partially positive H atom. On the other hand, the extra electron is localized only on the metal atom, which makes its electronic configuration a highly stable closed shell. When considering small metal clusters (3 atoms with a total charge (−1)), it turned out that the interaction is similar, but the hydrogen bonds between the metal and the pterin are weaker. A possible reason for this is the distribution of the negative charge all over the metal cluster rather than its localization on a single atom. It was also shown that the formation of non-conventional H bonds does not affect the ability of pterins to form conventional H bonds between themselves and form dimers and tetramers. In this case, the bond with the metal atom turned out to be slightly stronger than the bond between pterins.

The surface-enhanced Raman scattering (SERS) method is based on the interaction of metal with organic molecules, where a significant increase in the Raman effect occurs on an enhancing metallic substrate. Thus, Smyth et al. [161] used a silver colloid to detect xanthopterin, isoxanthopterin, and 7,8-dihydrobiopterin. Moreover, this method is able to distinguish between two geometric isomers of xantopterin with the same composition. The limit of detection (LOD) for pterins was 500 ng ml^−1^. The authors said that LOD can be improved, but this detection method itself has a significant advantage over other methods such as HPLC because it requires a very small amount of the sample and a short exposure time. Thus, this method is a rapid detection technique.

In 2022, an article was published where DFT calculations prompted the idea of using silver colloid for the Raman detection of pterin [162]. It has been supposed that SERS detection of pterin is better performed at pH > 8 since the deprotonated pterin Raman spectrum undergoes more dramatic changes upon the addition of silver compared with the neutral pterin. 

In addition to silver, nanostructured gold is often used for surface modification and SERS. The article by Castillo et al. [163] showed how Cap and gold-capped nanopillars interacted. A comparison of the SERS spectra and DFT calculations demonstrated that Cap mainly interacts with gold through the nitrogen of the amino group.

Based on the metal nanostructures, systems for the detection of pterins and their derivatives, in particular folic acid, are being developed. There exist numerous studies dedicated to interactions of folic acid with metals. Metal nanoclusters, especially silver and gold ones, have been actively studied in recent decades due to their outstanding properties: sub-nanometer size, high quantum yield fluorescence, controlled excitation and emission wavelengths, and biocompatibility due to biopolymer matrices. Thus, a method for the selective detection of folic acid based on fluorescent silver nanoclusters has been proposed [164]. The detection method is based on the fluorescence quenching effect in the presence of folic acid in solution; the detection limit was reported to be 0.032 nM.

A slightly more complex application of the effect of fluorescence quenching of a gold nanocluster has been proposed by H. Li et al. [165], where gold clusters have been obtained using the bovine serum albumin (BSA) protein. Folic acid, as in the example above, quenched the fluorescence of the clusters. However, when the complex interacted with the folate receptor of the cancer cell, fluorescence appeared again. The same static quenching of the fluorescence of a gold nanocluster on D-Trp upon interaction with folic acid has been observed [166]. The development of folic acid sensors using bimetallic Ag/Au fluorescent nanoclusters on AMP [167] and BSA [168] matrices with a quenching effect and detection limits of 0.109 µM and 0.47 nM, respectively, have also been reported. Larger non-fluorescent gold nanoparticles have been synthesized and modified with folic acid [169]. The potential application of these complexes in plasmonic and laser photothermal therapy for the selective targeting and damaging of cancer cells with an overexpression of folate receptors has been investigated. Therefore, single metal atoms, metal nanoclusters, and nanoparticles interacting with pterins opens a wide range of effects that have potential bioimaging, biophotonics, and biosensing applications. 

**Figure 10:**
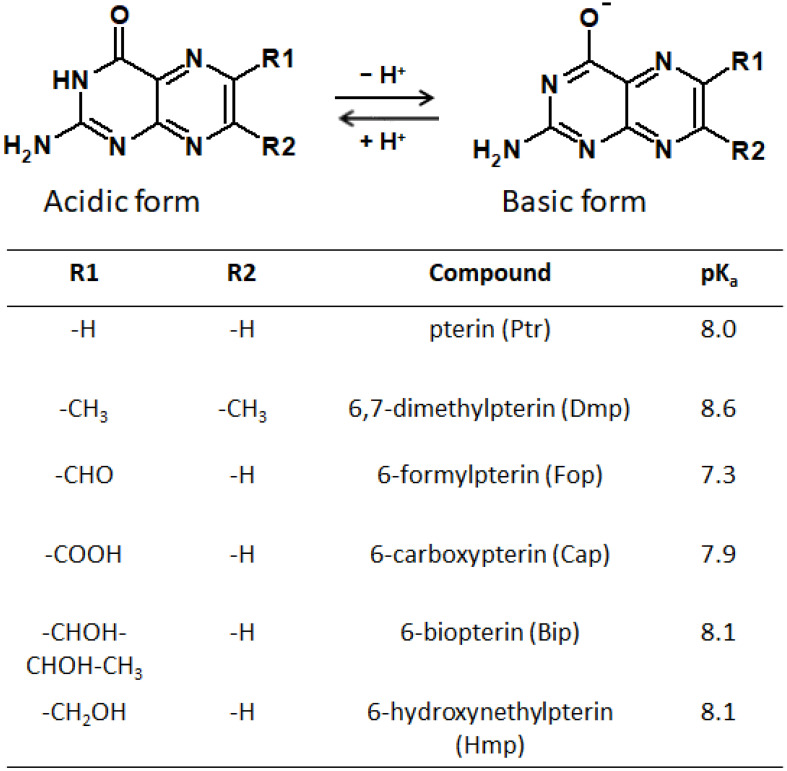
Structure and pK_a_ values for acid-basic equilibria of oxidized pterins in aqueous solutions [172].

It should be taken into account that pterin molecules can be in four forms depending on the protonation state: doubly protonated, singly protonated, neutral, and deprotonated [170]. At the physiological pH, the neutral and anionic forms predominate (Figure 10). The nature of the side substituent also significantly affects the photophysical and photochemical properties of pterins [171,172]. Thus, among other things, the objectives of our review include an analysis of how the nature of the side substituent affects the physical properties of pterins.

The photochemistry and photophysics of oxidized pterins have been studied in great detail. All oxidized pterins possess a high photochemical activity, and its main features are as follows:

1. Oxidized pterins possess a high fluorescence yield [173]. This gives a potential for their usage in bioimaging.
2. All oxidized pterins are able to form triplet excited states with a long lifetime [174,175].
3. Pterin triplets efficiently participate in electron transfer as acceptors [16,20].
4. Pterin triplets possess a high photosensitizing activity: they efficiently transfer energy to molecular oxygen and even biopolymer, inducing, for example, photoadducts and cyclobutane dimers of DNA [18,176]. The participation of oxidized pterins in photosensitized oxidation reactions will be discussed in detail in one of the next subsections.

**Figure 11:**
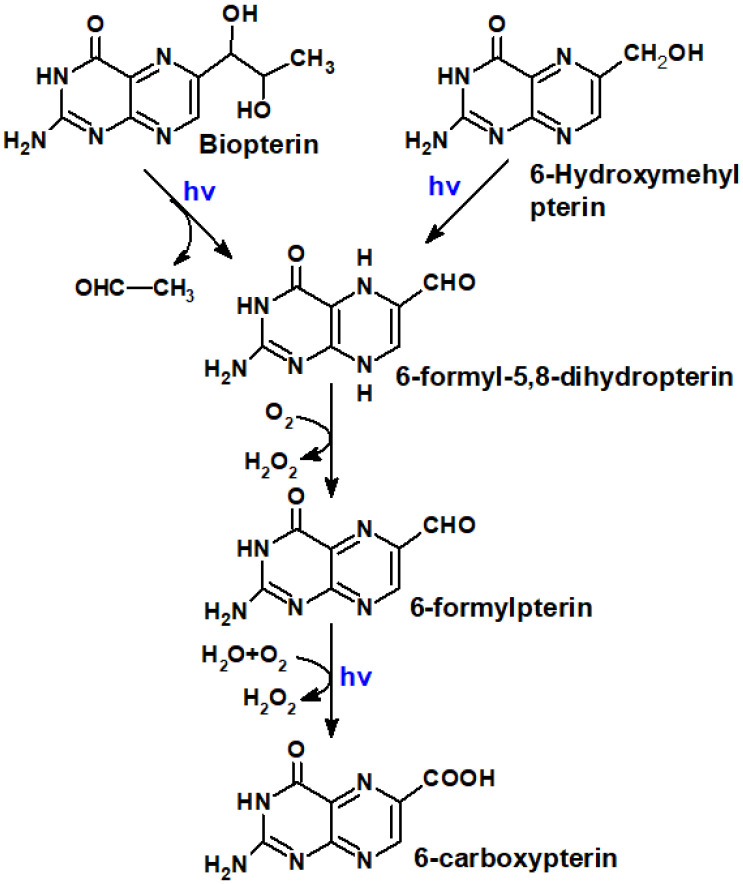
6-Biopterin and 6-hydroxymethylpterin photo-oxidation scheme [177].

Most of the oxidized ptrerins are formed as a result of coenzyme oxidation [29]. Bip, in its turn, is oxidized to Cap and Fop [Cabrerizo et al., 2004] (Figure 11).

### 5.1. Mutual Phototransformations of Pterins

**Figure 12:**
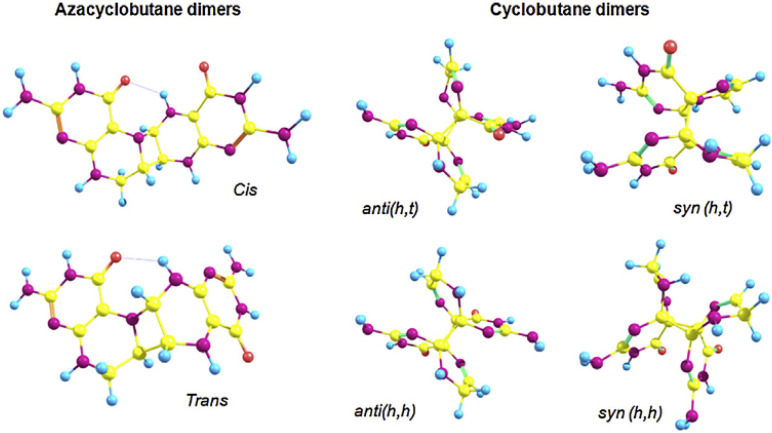
Cyclobutane and azacyclobutane isomers of (H_2_Ptr)_2_ [29]. © 2022 Elsevier.

Under the action of UV radiation in the presence of O_2_, dihydropterins can be oxidized to oxidized forms, or in the absence of O_2_, they can form dimers (Figure 12) [23,24]. The cis-azacyclobutane isomers of H_2_pterins are the most preferable isomers to be formed, which was shown by quantum-chemical calculations and mass-spectrometry [29].

**Figure 13:**
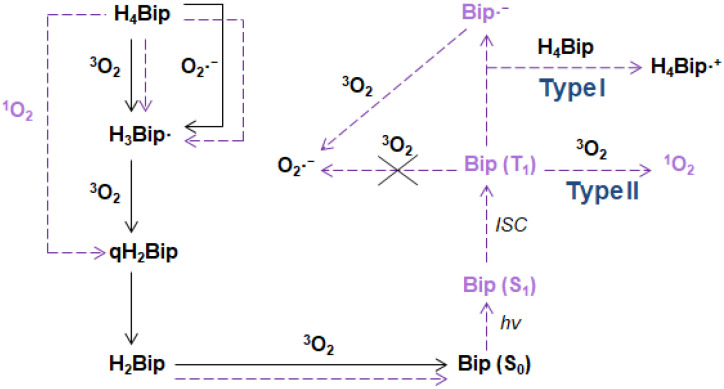
The scheme of H_4_Bip autoxidation (solid lines) and photooxidation (dashed lines) [30]. © 2022 Taylor and Francis.

H_4_pterins, in their turn, are oxidized under UV to H_2_pterins. Oxidized pterins play the role of photosensitizers during that process (Figure 13). The photooxidation occurs both according to a type I (direct electron transfer) and a type II mechanism (with participation of ^1^O_2_) [26,30]. As a whole, the process of H_4_pterin oxidation possess an autocatalytic chain-radical character. Moreover, a high reduction potential of H_4_pterins makes them favorable to participate in photoinduced electron transfer as donors [178] both in aqueous solutions and in vivo.

Regarding oxidized pterins, they are transformed according to Figure 11: Bip (Hmp) → H_2_Fop → Fop → Cap. The oxidation of oxidized pterins occurs much slower and with much less quantum yield when compared to H_4_pterins.

### 5.2. Absorption Spectroscopy of Pterins

**Figure 14:**
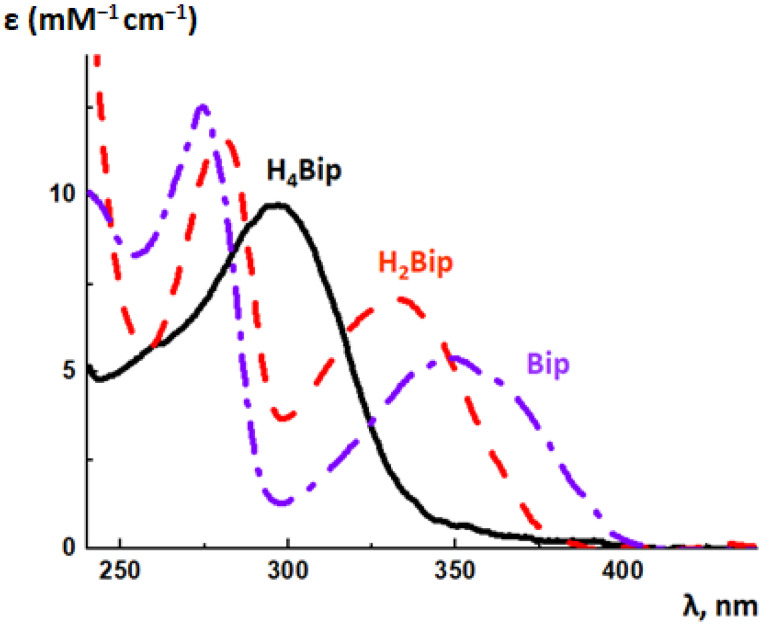
Absorption spectra of 5,6,7,8-tetrahydrobiopterin (H_4_Bip), 7,8-dihydrobiopterin (H_2_Bip) and biopterin (Bip) [179].

The photochemistry of dihydro- and oxidized pterins has been investigated in detail, whereas the photonics of H_4_pterins still remains almost unstudied. This is because of two factors. Firstly, the long-wave maximum in the absorption spectrum of H_4_pterins is blue-shifted (Figure 14). In living cells, UV is scattered by nucleus, organelles, and proteins; DNA and proteins can shield the pterin absorption. Secondly, reduced pterins are unstable, as they are subjected to oxidation by molecular oxygen. The latter circumstance greatly complicates the study of their photochemistry. 

The long-wave maximum in the absorption spectrum of acidic H_4_Bip is located at 298 nm in the neutral aqueous solutions; there is also a short-wave inflection at 260 nm (Figure 14). Since H_4_pterins are widely distributed in the tissues of higher organisms, they can act as targets of nonspecific UV. Reduced pterins neither fluoresce nor phosphoresce [174]. Oxidation of pterins can be observed through the change of their absorption spectra in the UV region (Figure 14). For example, during the oxidation of H_4_Bip, the absorption maximum shifts to the ultraviolet-A region. This is due to the fact that the long-wave maximum in the H_2_Bip absorption spectrum is located at 330 nm, and the long-wave maximum of the Bip absorption spectrum is located at 346 nm. 

**Figure 15:**
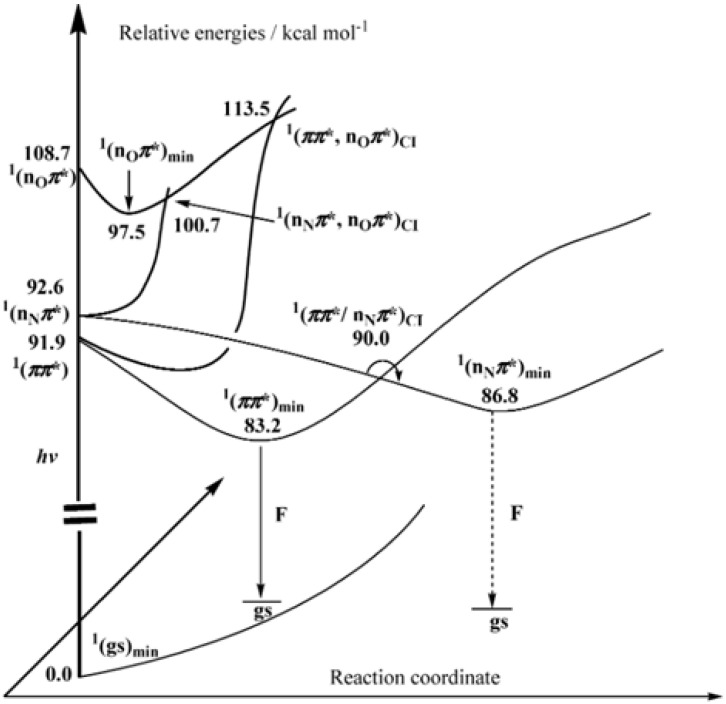
Relative energy profiles for the low-lying excited states (*) of acidic pterin [139]. © 2022 American Chemical Society.

**Figure 16:**
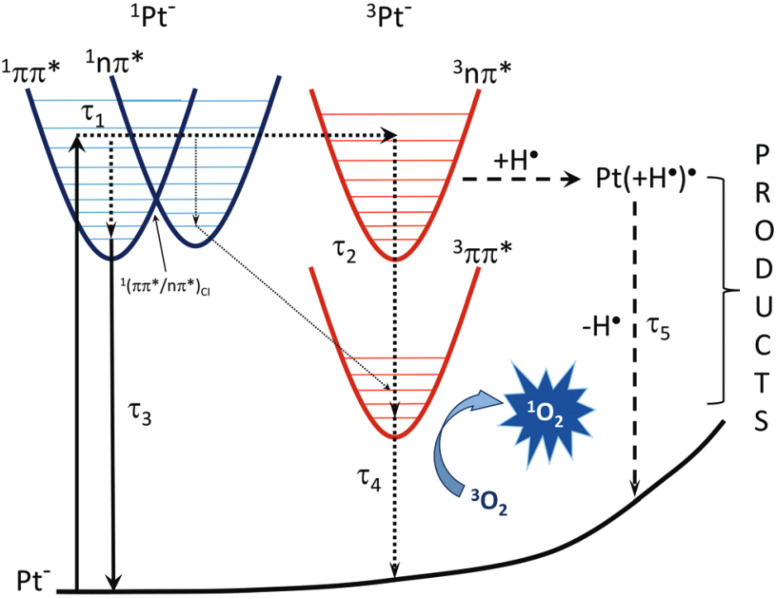
Relaxation pathways of Ptr^−1^ in aqueous solutions. Solid lines indicate radiative transitions, dashed lines—hydrogen transfer pathways, while the dotted lines indicate nonradiative decay transitions [180]. © 2022 Royal Society of Chemistry.

In the last few years, the interest has shifted from experimental studies to the quantum-chemical simulation of pterins’ absorption spectra. Chen and colleagues have conducted the first significant research in this area, in which they were the first to highlight the major excited state properties of pterins [139] (Figure 15). Photophysical properties have been studied with DFT and CASSCF, CASPT2 ab initio methods. The solvent effects on the low-lying states have been estimated by the polarized continuum model and combined QM/MM calculations. Two intense absorption transitions of the π→π* nature populate the ^1^(ππ*L_a_) and ^1^(ππ*L_b_) excited states. The ^1^(ππ*L_a_) state is exclusively responsible for the experimental emission fluorescence. The first ^1^(nNπ*) state can participate in pterin photophysics through the ^1^(ππ*L_a_/nNπ*) conical intersection. The internal conversion of ^1^Ptr* to the S_0_ state possesses an energy barrier of 13.8 kcal mol^−1^ for the acidic form to reach the (S_1_/S_0_) conical intersection. DiScipio et al. reproduced these results. They simulated the absorption spectrum of the basic form of Ptr^−1^ and established the nature of the lowest excited states, S_1_ (^1^nπ*) and S_2_ (^1^ππ*) (Figure 16) [180]. The S_2_ state is populated since its oscillator strength is two orders of magnitude higher than S_1_. The authors reported the energies of vertical triplet states for the first time: the energy of the T_3_ (^3^ππ*) state is close to S_1_ and S_2_, whereas T_1_ (^3^ππ*) is lower than S_1_ and S_2_ by nearly 1 eV. T_2_ (^3^nπ*) lies nearly 0.4 eV above T_1_. The most significant point is the reproducibility of experimental values, which were best fitted by PBE0 functional, whereas both CAM-B3LYP and M05-2X overestimated the S_n_ vertical energies by 0.3–0.5 eV.

**Figure 17:**
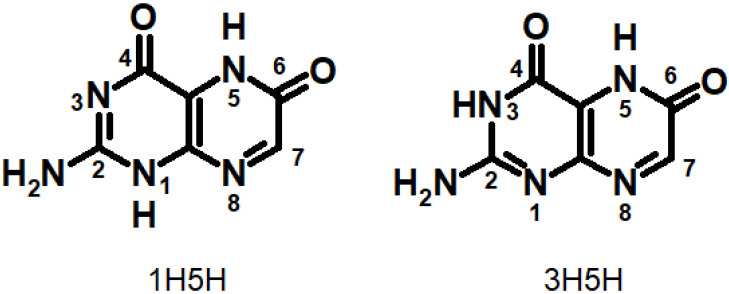
Chemical formulas of 1H5H and 3H5H xanthopterin tautomers.

Xanthopterin (Xap) is contained in the colored cuticle of the Oriental Hornet and it absorbs light and transforms it into chemical energy [181]. Roca-Sanjuan et al. studied the absorption and fluorescence spectra of different Xap tautomers computationally [182]. It was shown that the 3H5H tautomer (Figure 17) is the most stable one both in the gas phase and in solvent (well known for other oxidized pterins), which makes 3H5H responsible for the experimental Xap absorption spectrum. More interestingly, they evaluated electron and charge transfer in π-stacked Xap dimers. The electron donor-acceptor properties, the efficiencies of energy, and charge transfer of both 1H5H and 3H5H show more favorable characteristics than other tautomers for the electron donation of the neutral form and the electron attachment of the cationic system for energy and charge transport via π-stacking. 3H5H was predicted as the geometry with the most appropriate intrinsic features for light energy harvesting by Xap [182].

E. Wolcan has studied computationally the absorption spectra of pterin complexes with rhenium (Re) [141]. He has established that long-wave absorption is largely determined by metal-ligand charge transfer (MLCT), whereas ligand-metal CT and ligand-ligand transitions are highly energetic. The accuracy of electronic transition calculations is performed with the following order: PBE0 > B3LYP ≈ X3LYP > CAM-B3LYP for the Re(I) complex, whereas for the bare pterin it is B3LYP > PBE0. The redox properties, photophysical and, in particular, luminescent properties determine the interest towards Re-pterin complexes in sensor development [147,148]. Malcomson and Patterson have investigated the two-photon absorption (TPA) of pterins through the use of the quadratic response (QR) density functional theory [142]. Unconjugated pterins can be accessed by TPA through secondary states of both acidic (4.2–4.3 eV) and basic forms (3.6–3.9 eV). Conjugated pterins possess a larger number of states accessible with near IR. However, their long-wave accessible S_1_ states are ≈ 4.7 eV. Buglak et al. have made the first known attempt to study the absorption spectra of neutral H_4_pterin with TDDFT [178]. They have found that transitions to the Rydberg state predominate among the first six excited states. The optically bright π→π* S_0_→S_2_ and S_0_→S_6_ transitions have been correctly reproduced with B3LYP. Thus, it has been found that the S_1_ Ry state can solely be accessed with the excitation of the S_2_ (^1^ππ*) excited state. They have scanned the potential energy surface of H_4_Ptr along the C8aN1C2N3 dihedral (pyrimidine ring puckering) and found that the deactivation of the S_1_ state occurs without the energy barrier in a same way as with guanine, the excited state lifetime of which is estimated to be 0.3–0.5 ps [183,184]. Thus, the estimated excited state lifetime of H_4_Ptr has been nearly 0.5 ps.

Therefore, absorption spectroscopy has been studied in sufficient detail with TDDFT, *ab initio*, and TPA techniques. TPA opens the prospects for experimental manipulation with pterins aimed at physiological conditions of living cells. Evidently, the experimental time-resolved spectroscopy of H_4_pterins opens the prospects of metabolism photoregulation, especially when TPA is involved. H_4_pterins should be studied in more detail since their internal conversion is largely unknown yet still significant for the photoreception and photoregulation of metabolic reactions.

### 5.3. Luminescence 

The emission of oxidized pterins is realized as a result of excitation into the low energy band of 350 nm and shows a broad band centered at nearly 450 nm. The emission maxima of the basic forms are red-shifted by approximately 10 nm in comparison with those of the acidic forms [34]. The wavelengths of the fluorescence maxima (λ_F_) are listed in Table 1. The quantum yield of fluorescence (Φ_F_) and its lifetime (τ_F_) are lower at alkaline pH. The para-aminobenzoic acid residue acts as an internal quencher in conjugated pterins. As a result, Φ_F_ of folic acid and its derivatives is two orders of magnitude lower compared with unconjugated pterins [34]. Oxidized pterins possess a high quantum yield of fluorescence, largely affected by the nature of sidechain substituent (0.07 < Φ_F_ < 0.85). In contrast to oxidized pterin, the intensity of H_2_pterins fluorescence is lower by an order of magnitude (Table 1).

The quenching of S_1_ with O_2_ is negligible. However, the fluorescence is quenched by acetate and phosphate anions. Liu and colleagues established the nature of pH-related fluorescence quenching of pterin acidic form in the presence of acetate anions [186]. It occurs due to the excited state proton transfer (ESPT) from the amino group to one of the acetate oxygens. Liu and Sun have also studied the influence of pterin 6-substituent on the ESPT [187]. The substitution of 6-site with an electron-donating group (for example, dihydroxypropyl radical in biopterin) activates NH_2_ group, which makes it the favorable ESPT site. The introduction of an electron-acceptor group (in formylpterin) as the 6-substitute has inactivated the amino group and made N3 the preferable ESPT site.

The intense fluorescence of oxidized pterins makes their identification as impurities of folates and H4pterins (both are medical substances) possible even when the possibilities of chromatography are limited [26,175]. Furthermore, photodegradation of folates and H4pterins yields the oxidized pterins, and they intensify the photodegradation of folates and H4pterins with photosensitization reactions. Oxidized pterins are used for the fluorescent labeling of DNA due to their excellent optical properties and structural similarity to guanine [188]. Pterin labeling allows the study of DNA secondary structure as well as DNA-protein interactions [189]. The formation of thymidine-pterin adducts is possible photochemically, allowing exploitation of pterin absorption and emission properties for nucleic acid labeling [176].

Phosphorescence studies of pterins have been performed since the end of the 1970s. In a pioneer study by R.T. Parker et al., the phosphorescence of seven pterins (Cap, Fop, FA, Hmp, isoxanthopterin, Ptr, Xap) absorbed on filter paper has been measured both at room temperature and 77 K [190]. Impregnation of the paper with sodium acetate has significantly enhanced the pterin emission intensity. Most of the pterins, except for the isoxanthopterin, have shown both at room temperature fluorescence and delayed fluorescence emission. R.T. Parker et al. have concluded that the room temperature pterin phosphorescence has the potential of being used in analytical chemistry, but as we know from the past 40 years, fluorescence is mostly used during the separation of pterins.

C. Chahidi and co-authors were the first to study the emissive properties of pterin in aqueous solutions [9]. Both fluorescence and phosphorescence were pH-dependent. In an acidic pH, fluorescence is more intense than in an alkaline media, which has been shown for multiple pterin compounds (Table 1) [34]. However, C. Chahidi et al. established the two excited 3ππ* triplet states with the lifetimes equal to 0.3 µs and 2.3 µs. These lifetimes are in agreement with the later studies of A.H. Thomas’s group: 0.34 µs for 3Bip* [191]. For comparison, the lifetime of the S1 state is 9.1 ns for 1Bip* [192].

A. Krasnovsky Jr. et al. have studied the phosphorescence of Dmp and 6-arabopterin, or 6-tetrahydroxybutyl-pterin (TOP) at 77 K [174]. The quantum yields were equal to 2% and 6%, respectively. Phosphorescence lifetimes were 1.2 and 0.9 s, respectively. The S-T gap was found to be 0.45 and 0.42 eV, respectively. The maximum was located at 505 nm for both compounds, which is in agreement with the maxima of other pterins registered by R.T. Parker: 505 ± 10 nm [190]. Recently, A.H. Thomas with co-authors have measured the phosphorescence of a non-trivial pterin derivative 3-methyl-pterin, which has only a lactim form. Its phosphorescence lifetime was lower than of pterin: 0.86 s and 1.1 s, respectively [193].

In general, pterins possess intense spin-orbit coupling S-T intersystem crossing even in the absence of heavy metal or halogen atoms. That results in high quantum yields of ^1^O_2_ generation, which will be examined further in Section 5.4 and Section 5.5

### 5.4. Photosensitization Reactions

Oxidized pterins can absorb light and initiate photosensitizing reactions. Some authors classify sensitization mechanisms depending on which molecule the sensitizer interacts with [194]. The interaction of a sensitizer with a solvent or target molecule is referred to as type I sensitization. The interaction of a sensitizer with molecular oxygen is referred to as type II sensitization. Other authors divide sensitization mechanisms into type I and II, depending on whether charge transfer or energy transfer occurs [195].

All reactions associated with charge transfer are classified by these latter authors as a type I mechanism, and reactions associated with energy transfer are classified as type II. Therefore, the formation of a superoxide anion radical is referred to as type I and the formation of an electronically excited target molecule is of type II. In this regard, the mechanisms of photosensitized oxidation are generally classified into type I and type II as follows: reactions, in which free radicals of the target molecule or solvent are formed, are classified as type I; singlet oxygen formation reactions are classified as type II.

A transition from S_1_ to the T_n_ triplet excited state occurs through the intersystem crossing (ISC). The low rate of the S_1_/S_0_ internal conversion and the high rate of S-T ISC is a distinctive feature of sensitizer molecules. 

Triplet state pterins are involved in photochemical reactions, since their lifetime is ~1 × 10^−6^ s, whereas the lifetime of singlet excited states is ~1 × 10^−9^ s. For example, the lifetime of the Bip triplet state is 0.34 (±0.04) × 10^−9^ s [191], whereas the singlet state lifetime is 9.1 (±0,4) × 10^−9^ s [192]. The longer lifetime of triplet states is due to the spin-forbidden T_1_~>S_0_ transition.

The main photochemical reactions of oxidized pterins [16] include Reaction 8, which reflects the deactivation of the triplet state due to phosphorescence and intersystem crossing:

3Ptr* → Ptr(S_0_) (8)

The photochemical activity of the triplets is realized as: (1) the ability to transfer excitation energy; (2) the ability of an excited molecule to accept or donate an electron (as is known, both the donor and acceptor properties of molecules increase in an excited state). In addition, autoionization reactions (the interaction of two pterin molecules) are possible. In particular, the interaction of a molecule in the triplet excited state and a pterin molecule in the ground state can be possible with the formation of free radicals (Reaction 9) [9]:

3Ptr* + Ptr → Ptr^•−^ + Ptr^•+^ (9)

Radical species can then react with each other:

Ptr^•−^ + Ptr^•+^ → 2Ptr (10)

Under conditions of high Ptr concentration and high irradiation intensity, the interaction of two triplet excited molecules is possible [196]:

3Ptr* + 3Ptr* → Ptr^•−^ + Ptr^•+^ (11)

Pterins are able to generate singlet oxygen through energy transfer to O_2_ (Reaction 12), this reaction belongs to the type II sensitization mechanism:

^3^Ptr* + O_2_ → ^1^O_2_ + Ptr (12)

The Ptr•− radical anion formed during Reaction 9 and 11 can react with molecular oxygen to form the O2•− superoxide radical anion (Reaction 13):

Ptr^•−^ + O_2_ → Ptr + O_2_^•−^ (13)

The electron-donor properties of the pterin triplets can reveal themselves in the ability to transfer an electron to molecular oxygen with the generation of O2•−. However, it was not known for sure whether the pterin triplets are capable of forming O2•− (Reaction 14) [16,140]. The quantum chemical calculations showed that electron transfer from the pterin triplet to molecular oxygen is impossible except for Cap and Fop at alkaline pH only [196].

^3^Ptr* + ^3^O_2_ → Ptr^•+^ + O_2_^•−^ (14)

In the presence of an electron donor (D), the above reactions are accompanied by the reaction between ^3^Ptr* and D (Reaction 15). As a result, Ptr^•−^ and the D^•+^ donor radical cation are formed. This reaction belongs to the type I sensitization mechanism:

^3^Ptr* + D → Ptr^•-^ + D^•+^ (15)

The reverse electron transfer to the D^•+^ radical cation (Reaction 16) is the main reaction for the pterin radical anion in the absence of molecular oxygen and other electron acceptors:

Ptr•− + D•+ → Ptr + D(16)

**Figure 18:**
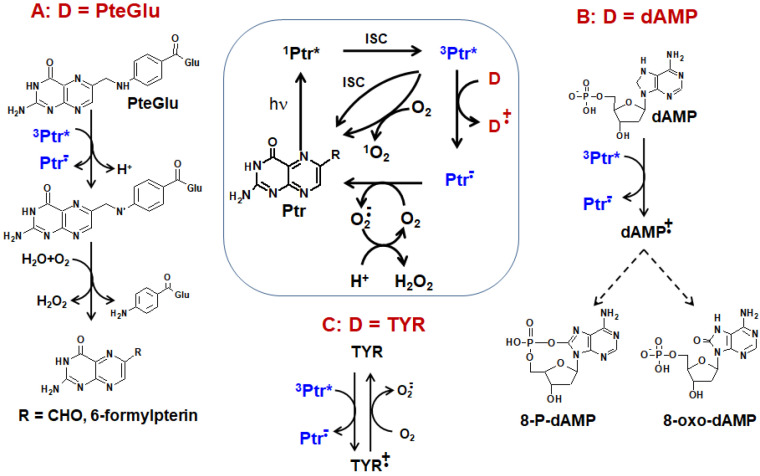
Schemes of pterin-sensitized electron donor oxidation. (**A**) folic acid (PteGlu) photooxidation; (**B**) Deoxyadenosine monophosphate (dAMP) photooxidation; (**C**) tyrosinase (TYR) inactivation [17,203].

The electron acceptor properties of pterin triplets are revealed during Reaction 15. Various compounds can act as electron donors in this reaction: amino acids [197,198] and proteins [199], nucleotides [191] and nucleic acids [200], lipids [201], as well as other biomolecules, in particular, both conjugated [202] and unconjugated pterins [26,30] (Figure 18). It should be mentioned that enzymes can be inactivated by the pterin triplets. For example, it has been shown that electron transfer from tyrosinase to ^3^Ptr* leads to enzyme inactivation [203] (Figure 18C).

The ability of pterins to oxidize biomolecules (primarily nucleic acids and proteins) is intensely used in photodynamic therapy (PDT) [204,205]. However, the main feature of a photosensitizer is the ability to generate singlet oxygen, which can be achieved by pterins with a high quantum yield of up to 50% [138].

### 5.5. Photogeneration of Singlet Oxygen

Oxidized pterins are efficient photogenerators of singlet oxygen (^1^O_2_). For example, Dmp and Top possess the quantum yields of ^1^O_2_ generation (Ф_Δ_) equal to 16% and 20% in air-equilibrated water solutions [14]. The nature of the side substituent has a significant effect on the quantum yield of ^1^O_2_ generation [172]. Ф_Δ_ of the acidic form of 6-hydroxymethylpterin (Hmp) is 15%, pterin (Ptr)—18%, 6-carboxypterin (Cap)—27%, 6-biopterin (Bip)—34%, 6-formylpterin (Fop)—45% [206]. These values rise to 21%, 30%, 37%, 40%, and 47%, respectively, in alkaline conditions. 

**Figure 19:**
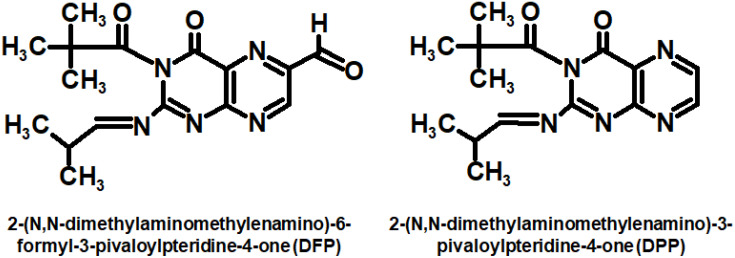
Synthetic pterins used for PDT [208].

In recent years, attempts have been made to use oxidized pterins for PDT of cancer. In particular, the photodynamic effect of using pterins has been studied on cancer cell lines. Along with 6-formylpterin and pterin [207], the synthetic analogues were used [204]. Currently, developments in this direction are ongoing. An increase in the permeability of cell membranes for pterins can be achieved by attaching nonpolar substituents to the pyrimidine ring (Figure 19) [208]. However, the addition of an extended alkane chain to the pyrazine ring is also beneficial [209]. Pterins can be applied for antimicrobial PDT (aPDT), especially when used along with other photosensitizing agents, for example, methylene blue [205].

But how do side substituents affect the quantum yield of ^1^O_2_ generation? The answer to this question will allow us to produce new pterin photosensitizers with improved properties and high ΦΔ. In this regard, an attempt was made to find molecular parameters, or descriptors, determining the sensitizing activity of pterins. To do this, a quantitative structure-property relationship (QSPR) analysis of the ability of pteridines to generate ^1^O_2_ was performed. QSPR and machine learning are used in photochemistry to offer fruitful results and allow for the prediction of the maximum absorption wavelength [210,211,212], fluorescence intensity [213,214], photoinduced toxicity, photolysis rate constant, photolysis half-life, and quantum yield [215,216,217]. In addition to pterins, the analyzed dataset included flavins and lumazine. Flavins are more efficient generators of singlet oxygen than pterins. It is possible that high values of ΦΔ are due to the presence of a carbonyl at the C2 position of flavins instead of an amino group in pterins. This assumption is confirmed by the fact that lumazine, which also has a carbonyl group at the C2 position, possesses the high quantum yield of ^1^O_2_ generation (ΦΔ = 44%) [218], whereas pterin has a ΦΔ equal to 18% [206].

The role of side substituents in ^1^O_2_ production by 29 pteridine compounds, including pterins, flavins, lumazine, and folates, has been analyzed. It has been found that a higher HOMO energy and electronegativity lead to a higher quantum yield of ^1^O_2_ generation [135]. Therefore, the oxidation potential of the side chain is an important factor determining the efficiency of ^1^O_2_ generation. The minor descriptors of the ground state dipole, dipole density, and electrostatic charge at N3 position also influenced Φ_Δ_ (all inversely correlated). The significance of N3 for ^1^O_2_ generation was further demonstrated experimentally: the addition of lipophilic decyl chain to N3 of pterin enhances the Φ_Δ_ from 18% up to 37% [138], whereas the alkylation of O4 increases it up to 50%. Thus, the alkylation of the pyrimidine ring allows for not only the improvement of the permeability of cell membranes but also enhances the efficacy of ^1^O_2_ generation.

### 5.6. ^1^O_2_ Quenching by Pterins

It is known that dihydropterins are effective quenchers of reactive oxygen species (ROS). Dihydropterins possess high rate constants of ^1^O_2_ quenching ($k_{\Delta }^{t}$): for example, $k_{\Delta }^{t}\;$ = (3.7 ± 0.3) × 10^8^ M^−1^ s^−1^ for H_2_Bip, (2.1 ± 0.2) × 10^8^ M^−1^ s^−1^ for H_2_Fop, (1.9 ± 0.2) × 10^8^ M^−1^ s^−1^ for Sep, (4.6 ± 0.4) × 10^8^ M^−1^ s^−1^ for H_2_Nep, and (6.8 ± 0.4) × 10^8^ M^−1^ s^−1^ for H_2_Xap. On average, these values fall by two orders of magnitude upon H_2_pyrazine ring oxidation: $k_{\Delta }^{t}$ = (2.9 ± 0.3) × 10^6^ M^−1^ s^−1^ for Ptr, (2.4 ± 0.3) × 10^6^ M^−1^ s^−1^ for Bip, (1.4 ± 0.2) × 10^6^ M^−1^ s^−1^ for Fop, and (2.3 ± 0.4) × 10^6^ M^−1^ s^−1^ for Nep (Table 2) [172]. 

The rate constants of ^1^O_2_ quenching by 15 pterins accompanied by 26 other heterocyclic compounds have been analyzed in a QSPR study [219]. The numbers of ammonium groups (aliphatic) and aromatic hydroxyls have been established as the most influential descriptors, both being inversely correlated with the logarithm of $k_{\Delta }^{t}$.

An attempt to evaluate the $k_{\Delta }^{t}$ value of H_4_Bip gave 5.4 × 10^8^ M^−1^ s^−1^, which is higher than that of H_2_Bip (3.7 × 10^8^ M^−1^ s^−1^) and similar to the rate constants of other reducing agents: ascorbate (3 × 10^8^ M^−1^ s^−1^), NADH (4.3 × 10^8^ M^−1^ s^−1^), and glutathione (9.4 × 10^8^ M^−1^ s^−1^) [179]. Therefore, the rate of ^1^O_2_ quenching largely depends on the oxidation state and has the following order: H_4_pterins > H_2_pterins ≥ oxidized pterins.

### 5.7. H_4_pterins as Photoprotectors

H_4_pterins can produce free radical species in the presence of molecular oxygen [30,118] and for this reason cannot be accounted as photoprotectors in true sense. However, if one can eliminate oxygen molecules, H_4_pterins can effectively dissipate UV excitation energy through internal conversion. Vibrational relaxation occurs primarily through the C8a-N1-C2-N3 dihedral angle. Based on the results of TDDFT modeling, the S_1_ state lifetime (τ_fl_) was estimated to be nearly 500 fs [179].

On the other hand, under UV irradiation, H_4_pterins can participate in photoinduced electron transfer as efficient electron donors [178]. According to quantum-chemical calculations, the vertical ionization potential of H_4_Hmp is equal to 6.8–7.3 eV for the gas phase [126,178] and to the slightly greater value of 7.2 eV for the water environment [178].

High electron-donor properties, low ionization potential of the ground state, and the S_1_ excited state (2.7 eV) make the photoinduced electron transfer from H_4_Hmp to an electron acceptor very likely. The electron transfer is not feasible in the gas phase but can occur in a polar environment [178]. Also, one should take into account that the conformation of the cation-radical form of 6-substituted H_4_pterin may differ from the conformation of the neutral molecule, which may be important with regard to the photoreceptor properties of 6-substituted H_4_pterins [179].

As can be seen from the analysis of published data, the photochemistry of oxidized pterins has been studied in sufficient detail, while the photochemistry of tetrahydro-reduced pterins has not been studied to the same extent. This is due, firstly, to the fact that reduced pterins are unstable in the presence of oxygen, which greatly complicates the study of their photochemistry. Secondly, H_4_pterins absorb light in the ultraviolet-B region (280–320 nm), which practically does not reach the Earth’s surface [220].

## 6. Biochemical and Physiological Application of Pterin Photochemistry

### 6.1. Evidence of Pteridine Participation in Photoreception

Flavins, or benzopteridines, are the nearest “relatives” of unconjugated pterins. Among pteridines, flavins are the most widespread photosensor molecules primarily because of the flavin adenine dinucleotide (FAD) and flavin mononucleotide (FMN) cofactors involved in light, oxygen, and voltage (LOV) blue light sensing using flavins (BLUF) domains containing photoreceptor proteins. Also, FAD is the main chromophore of the cryptochrome photolyase family (CPF) proteins. 

The common structural features of pteridines and flavins determine the similarity of the electronic structure and chemical properties of their excited molecules. The photochemical properties of flavins are determined by the presence of an isoalloxazine (2,4-dioxo-benzo-[g]-pteridine) ring within a developed system of conjugated double bonds (Figure 1), which allows the formation of stable radicals. In flavins (FMN or FAD), the light absorption band corresponding to the lower singlet level (S_1_) of excitation is in the blue region of the spectrum and has a maximum at 450 nm. The other two bands have an absorption maximum at 260 and 365 nm. The absorption of a photon increases the energy of the flavin by 265.8 kJ mol^−1^, making it a highly electrophilic excited molecule (Fl*). When an electron passes from a donor molecule to an excited flavin, a free radical (FlH· or Fl·^hich plays a key role in some flavin pho^) is formed, which plays a key role in some flavin photocycles (for example, the proposed mechanism of the BLUF domain, see below). The addition of one more electron transforms it into dihydroflavin (FlH_2_ or FlH^−^). In the dark, the photoreduced flavin undergoes oxidation, returning to its original state, which is a process that can proceed in a cyclic mode. The flavin radical can also be formed as a result of the oxidation of the photoexcited flavin dihydroform; this reaction is the basis for the functioning of DNA photolyases (see below) [221,222]. Additional stability of the flavin radical can be imparted by amino acids that surround the flavin in the reaction center of the protein [223,224].

The BLUF and LOV domains are minimal modules (about 100–110 a.a.) that are part of various regulatory proteins capable of perceiving and reacting to blue light [225,226]. The BLUF domain non-covalently binds the FMN or FAD chromophores. When light is absorbed, photoreceptor proteins with associated chromophores (flavins) undergo conformational changes that allow them to transmit signals to other proteins. The photocycles of BLUF photoreceptors are thought to involve PCET. 

**Figure 20:**
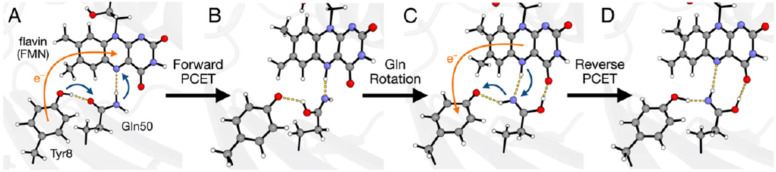
The photocycle of BLUF photoreceptor [228]. ©2020 National Academy of Sciences of the United States of America.

It is assumed that the photoexcited flavin takes an electron from the tyrosine molecule located in close proximity (at a distance of the hydrogen bond) in the reaction center, forming the flavin radical, FlH·. In this case, the proton (H^+^) is taken from the glutamine (Gln), which is also in close proximity to the flavin molecule (Figure 20). A redistribution of the H-bonding network rearranged in the reaction center occurs, which forces Gln to change its spatial orientation (Gln as an intermediate of a proton relay), which, in turn, leads to conformational changes in the photoreceptor and the formation of a protein signal form. The mechanisms of further signal transduction to acceptor proteins are not completely clear. The reverse process of electron transfer to tyrosine (PCET) leads to the restoration of the original form of the photoreceptor and the closure of the photocycle [227,228,229,230,231].

**Figure 21:**
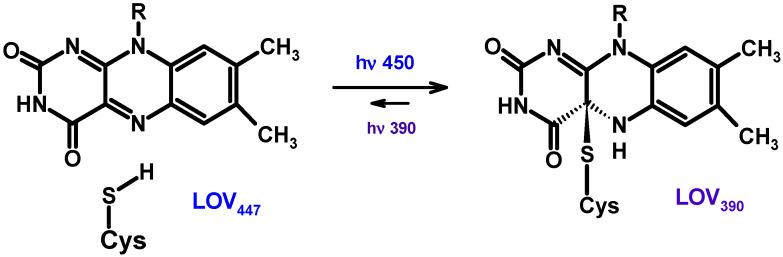
Тhe blue light-induced formation of a covalent adduct for LOV domains that thermally reverts to the parental state or can partially be photoreverted with UVA/violet light [225].

The photocycle of LOV domains begins with the dark state of LOV, in which FMN_ox_ is non-covalently bound to the protein. As a result of the photocatalytic process, a covalent bond is formed between FMN-C(4a) and conserved cysteine (LOV_390_ form) (Figure 21). 

It is assumed that the excited flavin passes into the triplet state, which leads to the formation of the FMNH·-H_2_CS·radical pair. FMNH· and H_2_CS· rapidly interact with each other and form an adduct. Since a covalent bond is formed, the time it takes for the reverse process to occur (from several seconds to several hours) depends on various factors: temperature, amino acids of the active center, etc. UVA/blue light can also accelerate this process [225].

The cryptochrome photolyase family (CPF) includes: DNA photolyases—enzymes that repair DNA damaged by UVB (280–320 nm) under the action of near UV and blue light (320–480 nm) [222,232,233,234] and cryptochromes—receptor proteins for near UV and blue light. Cryptochromes carry out photoregulation of transcription for various genes and also participate in circadian rhythms [232,234,235] and magnitoreception [236].

The main FAD chromophore is located in the active center of all CPF proteins. In DNA photolyases, flavin in the active center is in the FADH^−^ form. In cryptochromes, depending on the type of cryptochrome and the functions it performs, flavin can be found in almost any form: FADox, FAD·^−^, FADH·, or FADH^−^ [237,238]. FAD is responsible for substrate binding and basic photoreceptor function. The second chromophore performs the function of a “light-collecting antenna”, which captures additional light and transfers the excitation energy to FAD [234,239].

**Figure 22:**
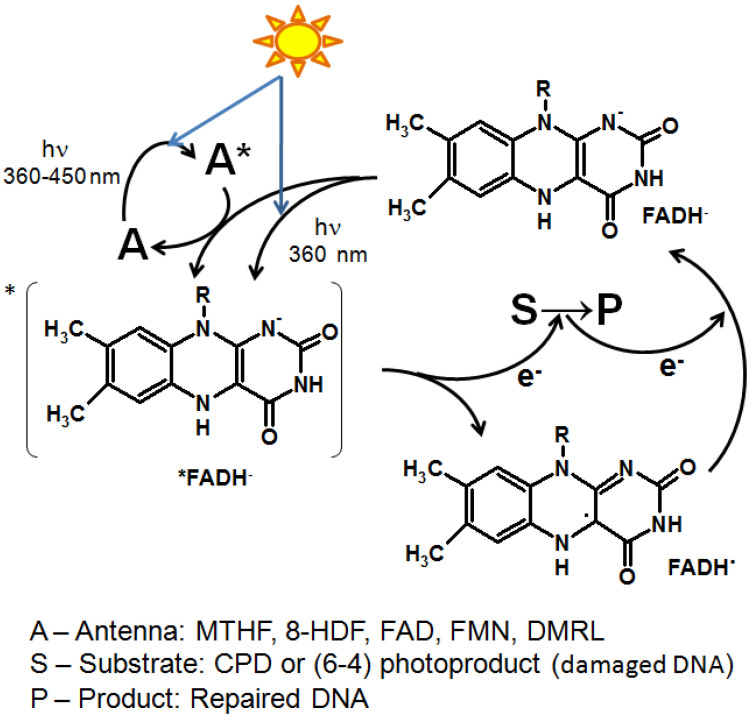
The photocycle of DNA photolyases.

The antenna molecule absorbs a UV-A/blue light photon (360–450 nm) and transfers the excitation energy by the Förster dipole-dipole resonance interaction to FADH^−^, forming a photoexcited *FADH^−^ molecule [240]. The latter can also be formed by direct irradiation of FADH^−^ in the region of the absorption maximum at 360 nm. The excited FADH^−^ then donates an electron to the substrate cyclobutane pyrimidine dimers (CPD) or pyrimidine-pyrimidone (6-4) photoproducts ((6-4)PP) to form FADH·. Furthermore, the electron density is redistributed within the damaged DNA molecule and the original structure is restored (Figure 22). After that, the electron returns to the flavin, regenerating the FADH^−^ form [232]. The photorepair quantum yield for CPD photolyases equals 0.7–1 (depending on the antenna and the efficiency of energy transfer from it to the flavin) and for (6-4) photolyases it is ca. 0.3 due to the more complex splitting mechanism of (6-4)PP [222,234,241].

The mechanism of functioning of cryptochromes is not fully understood yet. It is now generally accepted that the photocycle of cryptochromes involves the photoreduction of the FAD molecule, which is usually in a fully or partially oxidized state in the dark, to a partially or fully reduced form, which puts the protein into an active signaling state. Photoreduction can occur due to the transfer of an electron to the photoexcited flavin from the conserved aromatic amino acids, tryptophan and tyrosine, located in the active center of the photoreceptor. Aspartic acid can serve as a hydrogen donor [237,242]. At the same time, conformational changes occur in the protein structure, which are due to the redistribution of hydrogen bonds or a change in its surface charge. Also, phosphorylation of some amino acids allows cryptochrome to interact with other proteins with which it could not interact in the ground state [243,244].

**Figure 23:**
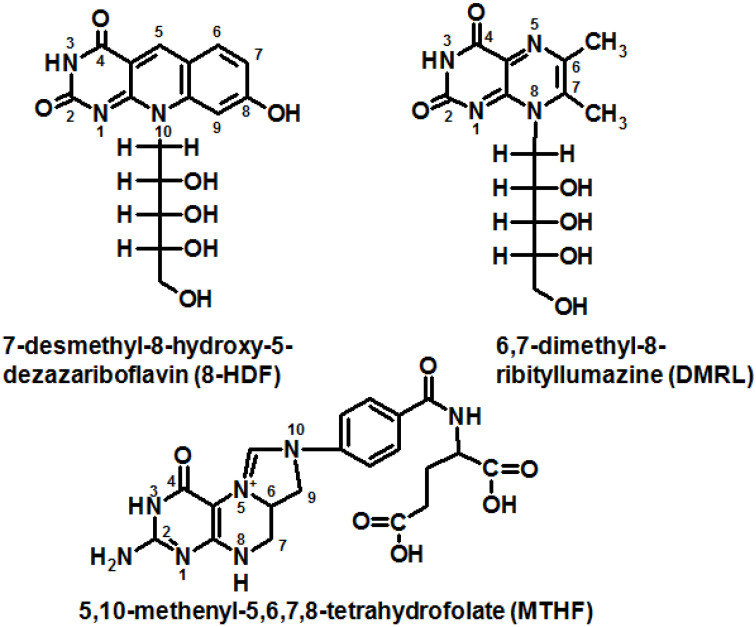
Antenna molecules of CPF proteins.

In CPF proteins, in addition to the main FAD chromophore, there are pigments (derivatives of flavins and pterins) that act as a “light-harvesting antenna” (Figure 23). Five molecules have been described as antennas of CPF [233]:

➢ 5,10-methenyl-5,6,7,8-tetrahydrofolate (MTHF) acts as an antenna in most eukaryotes and some prokaryotes [245,246];
➢ 7-desmethyl-8-hydroxy-5-deazariboflavin (8-HDF)—in some prokaryotes and protozoa eukaryotes, which have a biosynthetic pathway for this compound [247];
➢ FMN or the second FAD can also function as an antenna [248];
➢ it has recently been shown that 6,7-dimethyl-8-ribityllumazine can function as an antenna in some prokaryotic (6-4)-photolyases (Figure 23) [249].

The antenna chromophore has a higher extinction coefficient and a wider absorption band in the UVA region (ε 385 nm 25,000 M^−1^ cm^−1^ for MTHF or ε 440 nm 40,000 M^−1^ cm^−1^ for 8-HDF) compared with the flavin chromophore (FADH^−^ ε 360 nm 5600 M^−1^ cm^−1^) [221].

Until recently, there was no evidence of unconjugated pterin photochemical activity and its participation in photoreception. Only in the last few years has it been shown that some pterin compounds are involved in the reception of UV-B. It is assumed that H_4_pterins can act as chromophores of some UV photoreceptors [21,22]. The photoreceptors themselves have not yet been isolated and studied, however, it is known that H_4_cyanopterin (6-[1-(4-O-methyl-(α-D-glucuronyl)-(1,6)-(β-D-galactosyloxy]α-methyl-5,6,7,8-tetrahydropterin) (Figure 3) is responsible for the phototaxis of cyanobacteria in response to UV radiation. The photoreceptor containing cyanopterin has been shown to suppress negative phototaxis in response to UV and blue light [31]. The pgtA mutants (the pgtA mutant lacks the pteridine glucosyltransferase enzyme, which is related to the cyanopterin biosynthetic pathway) have the same positive phototaxis in response to red and green light as in the wild-type *Synechocystis* sp. PCC 6803. However, in response to the effect of white light, pgtA mutants are disoriented, cells move in a fan-like manner: in all directions with a slight positive phototaxis. A similar reaction is observed when exposed to UV and blue light. Notably, when *Synechocystis* sp. PCC 6803 is exposed to UV, cyanobacteria remain still, while pgtA mutants exhibit negative phototaxis [31].

Evidently, H_4_Bip is also a photoreceptor molecule: the action spectra of UV-B-induced anthocyanin accumulation in carrot cells indicated that H_4_Bip is involved in the regulation of anthocyanin synthesis [22]. In addition, the UV-B-induced activity of phenyalanine ammonium lyase (PAL), an enzyme that catalyzes the conversion of phenylalanine to ammonia and cinnamic acid, was suppressed by N-acetylserotonin (an inhibitor of tetrahydrobiopterin biosynthesis). The addition of H_4_Bip or Bip partially restored the UV-B-induced PAL activity in cells treated with N-acetylserotonin. It was assumed that there is a UV-B photoreceptor different from the UVR8 photoreceptor protein, in which the role of a chromophore is played by tetrahydropterin [22].

Thus, it has been established that H_4_pterins play the role of chromophores in certain UV-B receptors; however, these UV-B receptors themselves have not yet been isolated and studied. According to a hypothesis, the DASH cryptochrome is responsible for UV reception in cyanobacteria [21], whereas H_4_cyanopterin plays the role of a chromophore along with flavin [45]. The action spectrum of *Synechocystis* sp. PCC 6803 coincides with the action spectrum of the DASH cryptochrome. The action spectrum has three main peaks: the peaks at 300 nm and 380 nm correspond to pterins, the peak at 440 nm is characteristic for flavins. The peaks at 380 nm and 440 nm are also found in the fluorescence excitation spectrum of cryptochrome Ccry1 (the DASH cryptochrome of cyanobacteria) from *Synechocystis* sp. PCC 6803 [45]. If this hypothesis is not confirmed, we can assume that a new UV photoreceptor with H_4_pterin as a chromophore will be discovered in the near future.

As is known, H_4_pterins do not fluoresce and, therefore, cannot transmit a light signal by means of dipole-dipole energy transfer according to the Foster mechanism. H_4_pterins most likely do not form triplet forms and excited states with a long lifetime [179]. We assume that the transmission of the light signal occurs as a change of molecular conformation followed by structural changes in the UV-B receptor apoprotein similar to the UVR8, for example [250].

### 6.2. The Role of Pterin Coenzymes in the Photoregulation of Metabolism

**Figure 24:**
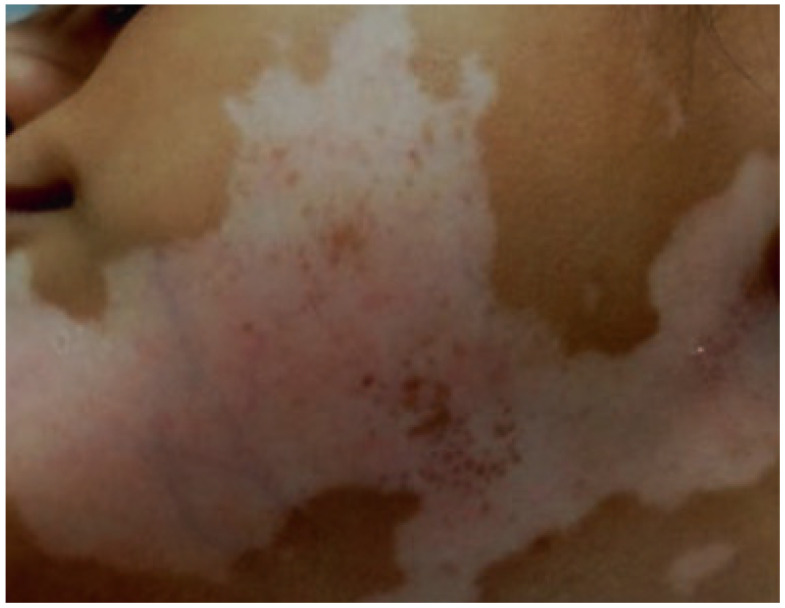
A clinical picture of vitiligo in a filipino individual [255]. ©2017 Springer.

We will assess the significance of pterin photochemistry for the metabolism regulation using vitiligo pathology as an example. Vitiligo is a pigmentation disorder, which is expressed in the disappearance of melanin and the appearance of depigmented skin areas (Figure 24). The etiology of vitiligo is still not known, but, it is believed to be associated with the metabolic functions of phenylalanine hydroxylase (PAH), an H_4_Bip-dependent enzyme of the initial stage of melanogenesis, and tyrosinase (EC 1.14.18.1)) [251,252], as well as with photochemical reactions of H_4_Bip and oxidized pterins [29,253]. Moreover, it has been shown that pterin can oxidize α-melanocyte-stimulating hormone [254].

**Figure 25:**
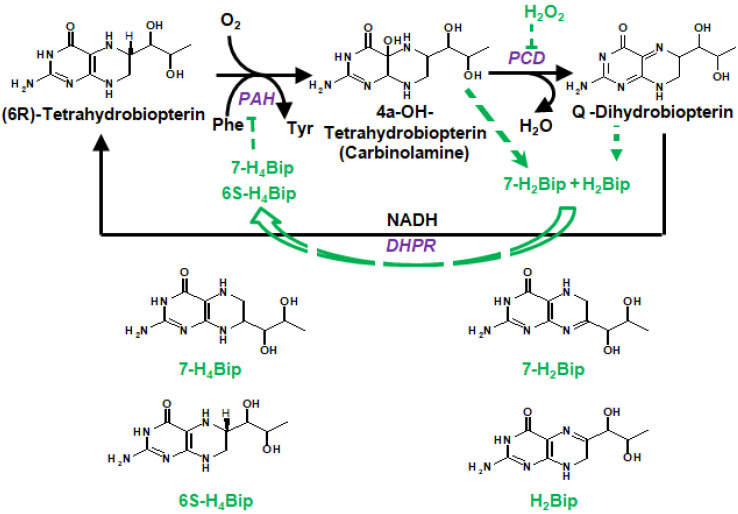
Disruption of the H_4_Bip regeneration cycle under oxidative stress and inactivation of phenylalanine hydroxylase [30] (black lines show normal H_4_Bip regeneration cycle, violation of the regeneration cycle is shown by green dotted lines). PAH—phenylalanine-4-hydroxylase; PCD—pterin-4a-carbinolamine dehydratase; DHPR—dihydropteridine reductase. 7-H_2_Bip—7-dihydrobiopterin, or dihydroprimapterin. © 2022 Taylor and Francis.

One of the most likely causes of vitiligo is a disorder of tyrosine (Tyr) metabolism (Tyr is a precursor of melanin). During the catalytic oxidation of Phe to Tyr, H_4_Bip is oxidized to 4a-OH-tetrahydrobiopterin (carbinolamine) (Figure 25). Dehydration of carbinolamine to quinoid dihydrobiopterin (qH_2_Bip) is catalyzed by pterin-4a-carbinolamine dehydratase (PCD) (EC 4.2.1.96). qH_2_Bip has a strong inhibitory effect on PCD, while 7,8-dihydrobiopterin (H_2_Bip) does not. In the absence of PCD, dehydration of carbinolamine proceeds non-enzymatically and leads to the formation of both H_2_Bip and 7-H_2_biopterin, or dihydroprimapterin [256]. 7-H_2_Bip, in contrast to 4a-OH-tetrahydrobiopterin, has an inhibitory effect on PAH [28]. Finally, the conversion of qH_2_Bip to H_4_Bip occurs with the participation of dihydropteridine reductase in a NADH-dependent reaction. Thus, the regeneration of H_4_Bip is necessary for the metabolism of phenylalanine, since: (1) a constant supply of H_4_Bip is required for the functioning of PAH; and (2) the accumulation of metabolites resulting from the non-ezymatic rearrangement of 4a-OH-tetrahydrobiopterin is unfavorable.

H_4_Bip directly regulates tyrosinase activity. The H_4_Bip binding site in tyrosinase has a sequence homologous to the H_4_Bip binding sites in PAH and PCD [27]. Under the low concentrations of Phe, H_4_Bip inhibits PAH [257]. In order to control tyrosinase activity by H_4_Bip, the presence of L-tyrosine is required. If L-DOPA acts as a substrate for tyrosinase, H_4_Bip has no inhibitory effect on the enzyme. H_2_Bip and Bip (products of H_4_Bip oxidation) do not have a significant inhibitory effect on tyrosinase, which means that the reaction of tyrosine hydroxylation to DOPA is controlled by the H_4_Bip/Bip ratio and can be initiated by H_4_Bip photooxidation [26,253]. It has been shown that H_4_Bip can function as a UV-B switch for de novo melanogenesis, since photoinduced oxidation of H_4_Bip can “remove” its inhibitory effect on tyrosinase [258].

It has been established that oxidative stress develops in vitiligo cells [259,260,261], and hydrogen peroxide accumulates at millimolar concentrations. Under oxidative stress conditions, tyrosinase is activated by low concentrations of hydrogen peroxide (<0.3 × 10^–3^ M), but is deactivated when the peroxide concentration is in the range of 0.5–5.0 × 10^–3^ M [262]. Under the oxidative stress conditions, the work of PCD can be inhibited by hydrogen peroxide, which leads to disruption of the H_4_Bip regeneration cycle [30] (Figure 25). The hypothesis that vitiligo is caused by a violation of the H_4_Bip regeneration cycle is one of the most developed and substantiated to date. Peroxide concentrations of less than 30 μM increase DHPR activity, but concentrations above 30 μM inactivate DHPR, which occurs through the oxidation of Met146 and Met151 protein sequences and leads to a disruption of the NADH-dependent active site of the enzyme [263]. PCD inactivation leads to nonenzymatic dehydration of 4a-OH-tetrahydrobiopterin, which proceeds with the formation of H_4_Bip and 7-H_2_biopterin [256](Figure 25). Enzymatic reduction of H_2_Bip and 7-H_2_biopterin with the participation of dihydropteridine reductase (DHPR) proceeds with the formation of 6(R,S)-5,6,7,8-tetrahydrobiopterin and 7(R,S)-5,6,7,8-tetrahydrobiopterin, accordingly, since DHPR has low stereospecificity [115]. The Michaelis constant for the interaction of (6S)-5,6,7,8-tetrahydro-L-biopterin (6S-H_4_Bip) and PAH is 2 times higher than for (6R)-5,6,7,8-tetrahydro-L-biopterin [264]; 7(R,S)-5,6,7,8-tetrahydro-L-biopterin (7-H_4_Bip) inhibits PAH [265] (Figure 25).

**Figure 26:**
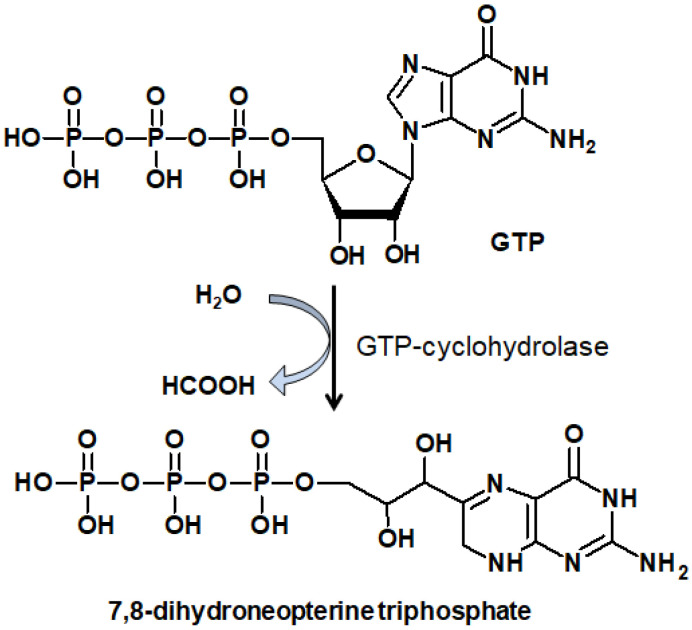
A simplified scheme of guanosine triphosphate (GTP) transformation to 7,8-dihydroneopterinphosphate, a precursor of H_4_Bip [28].

GTP-cyclohydrolase I (GTPCH) converts GTP to 7,8-dihydroneopterin-3’-phosphate (Figure 26), which is the limiting reaction for H_4_Bip biosynthesis. Inhibition of PAH (Figure 25) by the feedback mechanism leads to a three to five-fold increase in the activity of GTPCH. An increase in GTPCH activity leads to excessive de novo synthesis of (6R)-5,6,7,8-tetrahydrobiopterin [39,40]. Excessive synthesis of H_4_Bip, in turn, leads to the complete inhibition of tyrosinase [27], thus, tyrosine is not formed and, as a result, melanogenesis in epidermal cells stops.

The products of H_4_Bip oxidation, biopterin, and primapterin, accumulate in the depigmented epidermis cells (evidently, as a result of nonenzymatic oxidation of H_2_Bip and H_4_Bip by molecular oxygen) and exhibit characteristic fluorescence under ultraviolet irradiation [176,266]. Photolysis of biopterin under aerobic conditions leads to the formation of additional amounts of peroxide in vitiligo [199,267]. In addition to Bip and primapterin, epidermal cells accumulate 6-carboxypterin, a product of biopterin oxidation, which also effectively sensitizes the formation of ROS under UV exposure [34]. This circumstance makes H_4_Bip (H_4_Bip accumulates in depigmented skin as a result of excessive de novo synthesis) sensitive to UV radiation and leads to the addition generation of ROS sensitized by oxidized pterins—A “snowball” effect. The study of the process of photooxidation and photosensitized oxidation of H_4_Bip is significant for understanding the etiology and course of the disease, as well as for developing methods of vitiligo therapy [26,29,253].

### 6.3. Evolutionary Aspects of Pterin Photochemistry

Recently, it has become known that pteridines (pterins and flavins) are chromophores of photoreceptor proteins: photolyases, cryptochromes, etc. Pteridines can participate in redox processes and, on the other hand, function as ultraviolet and blue light receptors [268]. In the first instance, such wide functionality can be associated with their resistance to UV radiation. UV radiation and the blue part of the spectrum of the Sun were the most important sources of free energy during the period of pre-biological and early biological evolution on Earth. During this period, those substances that were available from abiogenesis were probably used. It has been shown that pterins and isoalloxazines (flavins) can be formed upon thermal condensation of abiogenic amino acids [269,270,271]. In the period of pre-biological evolution, chromoproteinoids containing pteridines (pterins and flavins) as chromophores could function as catalysts in dark redox reactions and as photosensitizers in photoinduced processes. The availability of this group of compounds may indicate their antiquity and possible participation at all stages of evolution.

The second important evolutionary aspect of pterin photochemistry is the pteridines and the “RNA world” [33,272]. The existing set of bases in nucleic acids is the product of evolution and selection. A variety of heterocycles could participate in the selection. It has been shown that pteridines are sterically suitable and can be inserted into nucleotide sequences [188]. Flavins and pterins could enrich the catalytic and photocatalytic capabilities of primitive polyribonucleotides lacking redox functions. Under conditions of abiogenesis, pteridines could absorb UV and function as photocatalysts of free radical processes leading to the synthesis of compounds for further pre-biological evolution [273]. Under non-oxidative conditions of pre-biological and early biological evolution, the structural similarity of pterins and purines may have allowed pterins to be incorporated into proto-RNA. In the absence of significant amounts of free oxygen in the atmosphere, H_2_pterins and H_4_pterins conjugated into proto-RNA could function as electron and hydrogen donors in various processes of the complication of carbon compounds on the way to life, including processes associated with the storage of free energy.

With the oxygenation of the environment and appearance of the ozone layer, the functions of pteridines also changed. The pyrazine part of the pterin structure became oxidized, and the π-electrons of its double bonds began to significantly affect the electronic configuration and redox properties of pterins. Oxidized pterins are dominated by the processes of fluorescence, S-T intercombination conversion, as well as the processes of energy transfer to molecular oxygen with the formation of singlet oxygen, which is dangerous for polyribonucleotides and other molecules. In this case, the main photoreception of UV energy and its storage as the reaction products have passed to more specialized chromophores—porphyrins, which possessed hydrophobicity, localization in membranes, and absorption of light in the visible part of the spectrum. At the same time, pteridines retained their UV chromophoric functions as a part of photoregulatory proteins. The catalytic redox functions of pteridines also appear to be conserved throughout the evolution.

Another evolutionary aspect of pterin photochemistry is the possibility of the participation of H_4_pterins in the photoprotection of cyanobacteria during the early stages of biological evolution. Prior to the Great Oxidation Event, oxygen was absent in the environment, whereas cyanobacteria were among the first living organisms on the Earth more than 2.4 billion years ago [274,275]. H_4_pterins are found in high concentrations (at a ratio of 1:1.6 towards chlorophyll a) in cyanobacterial cells [44]. Since H_4_pterins have high photostability and an ultrashort lifetime of the excited states along with low ionization potential [178], this makes them an ideal candidate for the role of photoprotectors and antioxidants.

## 7. Future Prospects

The physicochemical properties of pterins are largely influenced by their redox state: different pterin groups (oxidized pterins, H_2_pterins, and H_4_pterins) have different biomedical functions. Free-radical species and electronically excited states of pterins are also important in the context of biomedicine. However, so far the experimental techniques of their “manipulation” have been limited, although this does not prevent us from theoretical studies and discussions of future prospects. 

The UV wavelength needed to excite pterins lies in the region of 300–350 nm, which has a very poor tissue penetration because of the scattering from the cell nuclei, organelles, and the cellular surface [276,277]. The optical penetration into biological tissue is low. However, this problem can be solved since tissue penetration depends on the wavelength used. Longer wavelengths (>800 nm) appear to penetrate up to 4.2 mm [278]. The use of irradiation with λ = 600–1000 nm via two-photon absorption (TPA) increases tissue penetration and the spatial resolution of photoactivation [142]. Therefore, all pterin forms can be accessed with light in tissues and cells, and specific prospects beneficial for the development of new approaches in biomedicines arise.

### 7.1. Oxidized Pterins

The significance of the physical chemistry of pterins is determined by their biochemical functions. First, oxidized pterins are used in medicine as biomarkers of oxidative stress, cardiovascular diseases, neurotransmitter synthesis, inflammation, and immune system activation [74]. They are also used in diagnostics of diseases such as phenylketonuria [69], vitiligo [279], hyperphenylalaninemia [280], cancer [70,93], etc. Spectroscopic methods, along with noble metal nanostructures, can be used for the detection of oxidized pterins [162,163,281].

Second, oxidized pterins can be used as photosensitizers in anti-tumor and anti-bacterial PDT since they produce ^1^O_2_ with a quantum yield of up to 47% [206]. The addition of various nonpolar side chains allows for the improved penetration of pterin sensitizers into cells and increases PDT efficacy [138,208]. Pterins can be used for anti-tumor therapy even in the absence of light. A newly synthesized conjugated derivative of pteridine and benzimidazole was delivered to tumor cells using soft nanocarriers: micelles and liposomes. The conjugate was 37 times less toxic towards a normal cell line than towards a tumor [282].

Third, if one wants to improve the photodynamic properties of pterins, one should alkylate the pyrimidine ring to enhance both the lipophilicity, log D (from −0.16 up to 3.53), and Φ_Δ_ (from 18% up to 50%) [137]. Also, the change of 6-substituent to a more electronegative one allows for the increase of Φ_Δ_ [135].

The sensitizer is incorporated into a nanoparticle (NP) (with a size of 20–50 nm) in the last generation of photosensitizers (PS) [283]. Most of PSs are poorly soluble in water; therefore, insertion of PS into NP often improves the efficiency of PS delivery to the target cells. Inorganic (gold, silica, metal oxide, etc.), polymer, amphiphilic (micelles and vesicles), and metal–organic frameworks (MOF) based NPs are used nowadays for PDT [283]. Evidently, pterins can be used for the development of such 3rd and 4th generation of PSs, but obviously it is not required, and we have not found examples of such studies: many pterins are hydrophilic compounds that easily spread in biological fluids. Usually the purpose is the opposite: to improve adsorption of pterin on biomembrane and cell penetration. For this reason, the chemical modification of pterin photosensitizers with nonpolar radicals (for example, O-decyl-pterin synthesis [201]) improves the efficacy of pterin PS. Nevertheless, the conjugation of folic acid to a metal oxide photosensitizer, for example, is intensely used in cancer PDT [284]. The insertion of pterin sensitizers has not been developed yet (except a single study on pterin immobilization on silicon [285]). Future prospects are obvious in this case: one should develop methods of a self-assembly monolayer formation, allowing the design of photosensitive surfaces with potential microbiological self-cleaning properties.

### 7.2. H_2_pterins

H_2_pterins are intermediate metabolites: they are too unstable for diagnostics, yet not as important for metabolism as H_4_pterins. For this reason, urinary H_2_pterin level is not as indicative as serum H_2_pterin level and is used in diagnostics [286]. However, they do participate in the enzymatic reactions of pterin metabolism as substrates (for example, in the work of DHPR [56], 6-hydroxymethyl-7,8-dihydropterin pyrophosphokinase (HPPK) [287], etc). Evidently, these metabolic reactions are minor and related to the pterin metabolism itself and are not as important as the redox reactions of H_4_Bip or H_4_Nep. Therefore, their usage as biomarkers in medical diagnostics, photoreceptors, and photoregulatory molecules in phototherapy is quite limited. Potentially, H_2_pterins can be used as dietary supplements to stimulate H_4_Bip formation in a similar way to folic acid [288]. However, chirality issues arise in this case: DHPR possesses low stereospecificity and reduces H_2_Bip to both 6R-H_4_Bip and 6S-H_4_Bip. 6S-H_4_Bip inhibits PAH to some extent: upon interaction with 6S-H_4_Bip, Km of PAH decreases by half when compared to 6R-H_4_Bip [289]. Therefore, H_2_Bip is not ubiquitously used as a dietary supplement and medical substance.

### 7.3. H4pterins

Preparations of tetrahydrobiopterin are successfully used in the treatment of diseases associated with disorders in the genes encoding H_4_Bip biosynthesis or recycling (phenylketonuria, vitiligo, and a number of neurodegenerative and cardiovascular diseases). With its redox reactions, H_4_Bip is able to regulate the levels of reactive oxygen species in the endothelium. The possibility of using H_4_Bip as a therapeutical agent in cardiovascular medicine is intriguing [118].

Н4pterins are remarkable, first of all, as potential photoreceptor chromophores [21] and metabolic photoswitches [258]. On the one hand, they should actively participate in photoinduced electron transfer (PET) as electron donors. On the other hand, in anaerobic conditions, H_4_pterins can act as phototriggers, which change their conformation upon excitation, or even as photoprotectors. The latter was probably exploited by cyanobacteria before the Great Oxidation Event [290]. H_4_Bip can be responsible for aromatic amino acid synthesis photoregulation, NO synthesis regulation, melanogenesis, etc.

Moco is essential for the pathogenic bacteria *Mycobacterium tuberculosis* and *Pseudomonas aeruginosa* [53]. Moco can be affected in vivo by two-photon absorption provoking oxidation of the cofactor [49]. This idea is related to two-photon absorption usage in antibacterial therapy.

H_4_pterins should be used in the development of novel methods of pterin detection. Currently, this is achieved, in particular, using metal [291]. However, thorough development of these H4pterin detection methods is limited by their instability in the presence of oxygen. Therefore, novel detection approaches should possess fast pterin manipulation in the absence of oxygen.

## 8. Conclusions

The physical chemistry and photonics of oxidized pterins have been studied in sufficient detail. However, the physical chemistry of H_4_pterins has been poorly studied due to their chemical instability and susceptibility to oxidation by molecular oxygen. Meanwhile, the biochemistry of H_4_pterins as protein coenzymes has been studied to some extent due to their high importance for biology and medicine. Evidently, when bound to proteins, H_4_pterins are less susceptible to spontaneous oxidation by oxygen.

It is H_4_pterins that play the major role in photobiochemical processes: these compounds can be photoreceptor molecules (for example, H_4_Cyp in DASH cryptochromes [45]) and regulators of metabolic cascades, in particular melanogenesis [258,266]. In this regard, the physical and chemical properties of H_4_pterins should be beneficial for phototherapy [29,253].

To solve biomedical problems, it is also necessary to study the femtochemistry of H_4_pterins. We have come close to solving this problem and have shown that H_4_pterins: (1) have an excited state lifetime of nearly 0.5 ps, and (2) are able to effectively donate an electron as a result of PET [178].

Under anaerobic conditions, H_4_pterins can play the role of effective photoprotectors. This can explain their extremely high concentration in cyanobacteria (just 40% lower than in chlorophyll a [44]), which are the “authors” of the modern aerobic atmosphere. It has become apparent that the photoprotective H_4_pterins can be used in vivo in medicine and industry. Moreover, it has been shown that H_4_neopterin can be used as an X-ray photoprotector [292]. If the synthesis of oxidation-resistant H_4_pterins is established (for example, 5,10-methenyl-tetrahydrofolate is an oxidation-resistant H_4_pterin), they can also be used as a component of sunscreens.

Regarding oxidized pterins, the potential for their detection using metal nanostructures should allow for a LOD of several nM. Both silver and gold nanostructures have been established as potential tools [161,162,163]. The second aspect is the utilization of oxidized pterins in PDT both as a component of synergistic systems [205] and as a part of nano-sized delivery systems [282].

## Figures and Tables

**Table 1:** Wavelengths of fluorescence maxima (λ_F_), fluorescence quantum yields (Φ_F_), and fluorescence lifetimes (τ_F_) in air-equilibrated aqueous solutions of oxidized pterins [34] and H_2_pterins [185].

Compound	λ_F_, nm	Φ_F_	τ_F_
Ptr^0^ Ptr^−1^	439 456	0.33 0.27	7.6 5.0
Mep^0^ Mep^−1^	448 460	0.61 0.61	13.3 11.2
Hmp^0^ Hmp^−1^	449 457	0.53 0.46	11.0 8.4
Fop^0^	446	0.12	7.9
Fop^−1^	454	0.07	2.2
Cap^0^	439	0.28	5.8
Cap^−1^	451	0.18	4.1
Dmp^0^	433	0.85	13.5
Dmp^−1^	445	0.84	11.6
Bip^0^	441	0.36	9.1
Bip^−1^	455	0.29	7.6
Nep^0^	440	0.31	7.4
Nep^−1^	454	0.47	10.7
Rap^0^	441	0.47	10.7
Rap^−1^	455	0.40	7.5
H_2_Fop	528	8.7 × 10^−3^	0.34
Sep	533	7.0 × 10^−3^	0.28
H_2_Bip H_2_Nep	425 425	9 × 10^−3^ 5 × 10^−3^	0.30 0.31
H_2_Hmp	425	3 × 10^−3^	0.21
H_2_Mep	410	3 × 10^−3^	0.18

**Table 2:** Quantum yields of ^1^O_2_ generation and rate constants of ^1^O_2_ total quenching [172].

Compound	Φ_Δ_	$\;k_{\Delta }^{t},\;10^{6}\;M^{-1}\;s^{-1}$
Ptr^0^ Ptr^−1^	0.18 0.30	- 2.9
Cap^0^ Cap^−1^	0.27 0.37	- 1.4
Fop^0^ Fop^−1^	0.45 0.47	- 1.4
Bip^0^ Bip^−1^	0.34 0.40	- 2.4
Nep^0^ Nep^−1^	0.23 0.34	- 2.3
Mep^0^ Mep^−1^	0.10 0.14	- 8.0
Dmp^0^ Dmp^−1^	0.04 0.10	- 31
Rap^0^ Rap^−1^	0.13 0.16	- 3.6
Hmp^0^ Hmp^−1^	0.15 0.21	- 3.1
H_2_Fop^0^	0.001	210
H_2_Bip^0^	0.001	370
H_2_Nep^0^	0.001	460
H_2_Xap^0^	0.001	190
Sep^0^	0.001	550

## Data Availability

Not applicable.
